# Genome-wide association studies and genomic selection for leaf-related traits in maize

**DOI:** 10.3389/fpls.2025.1669346

**Published:** 2025-12-09

**Authors:** Xinyu Yang, Penghao Wu, Wentao Cui, Dilinigeer Alimu, Kaixiang Wang, Jiaojiao Ren

**Affiliations:** College of Agronomy, Xinjiang Agricultural University, Urumqi, Xinjiang, China

**Keywords:** maize, leaf length, leaf width, leaf number above the upmost ear, genome-wide association study, genomic selection

## Abstract

Leaf morphological characteristics are critical factors affecting plant architecture and canopy photosynthesis, all of which ultimately affect grain yield. Elucidation of the genetic basis of maize leaf-related traits could assist breeders in designing effective breeding strategies. Genomic selection (GS) is an effective method to accelerate the breeding process. We performed a genome-wide association study (GWAS) and GS for leaf-related traits in a natural maize population consisting of 291 inbred lines. The GWAS panel was phenotyped at four environments for the leaf length of the first leaf above the upmost ear (L1), the upmost ear leaf (L2), the first leaf below the upmost ear (L3), leaf width of L1, L2, and L3, leaf area of L2 (LAr2), and leaf number above the upmost ear (LNAE), and genotyped by sequencing. The heritability of leaf-related traits was ranged from 77.93% to 87.54%. A total of 24 unique significant SNPs were identified for leaf length at the *p*-value threshold of 2.968 × 10^–6^ by FarmCPU and BLINK models. The phenotypic variation explained (PVE) by each SNP ranged from 4.82% to 20.7%. A total of 34 unique significant SNPs were identified for leaf width, each with a PVE ranging from 0.01% to 17.2%. A total of 14 unique significant SNPs were identified for LAr2, each with a PVE ranging from 0.7% to 21.8%. A total of 19 unique significant SNPs were identified for LNAE, each with a PVE ranging from 1.21% to 25.01%. Eleven pleiotropic SNPs controlling leaf-related traits were identified, indicating that the leaf length and width at different leaf positions may be influenced by one or more common loci. A total of 122 candidate genes were retrieved, among which *Zm00001eb297330*, *Zm00001eb275550*, *Zm00001eb377180*, *Zm00001eb296310*, and *Zm00001eb178140* are key candidate genes for leaf-related traits. The results of GS indicated that a training population size of 70% and a set of 3000 SNPs were adequate for the application of GS in maize leaf-related traits. This study provides important reference information for further elucidating the genetic basis of leaf-related traits and applying GS in maize breeding programs.

## 1 Introduction

Maize (*Zea mays*) is one of the three major crops globally, playing a crucial role in food security and export trade (Erenstein et al., 2022). The concept of crop ideal type was first proposed in 1968, which is a plant model with high yield and high quality potential (Donald, 1968). Maize plant architecture encompasses plant height, ear height, tassel branch number, and leaf morphology related traits. Leaves are vital organs for photosynthesis, respiration, and transpiration (Lv et al., 2023). Structural features of leaves, such as leaf length, leaf width, leaf area, and leaf number, can significantly influence the rate of photosynthesis and carbohydrate accumulation by regulating light energy utilization (Niinemets and Sack, 2006; Yan et al., 2024). Among all maize leaves, the ear leaf and its adjacent upper and lower leaves (i.e., the three leaves surrounding the main ear) contribute the most significantly to yield (Chen et al., 1996; Bai et al., 2000). Approximately 50% of the carbohydrates accumulated in maize kernels originate from the upper third of the stalk’s leaves, while around 30% come from the middle leaves (Camacho et al., 1995). The leaves located above the main ear are relatively young and more metabolically active, while leaves located below the main ear are susceptible to shading and senescence (Shaver, 1983; Stewart et al., 1997). Maize kernel yield declines significantly when leaves above the main ear were removed (Lima et al., 2010). Currently, the breeding trend of maize leaves focuses on the synergistic enhancement of photosynthetic efficiency and stress resistance, aiming to develop excellent varieties characterized by upright and compact growth habits, consistently vibrant green foliage, and strong overall stress tolerance, in order to support the breeding goals of high yield, stable yield, and broad adaptability for maize. Thus, exploring the phenotypic variation and genetic basis of leaf length, leaf width, leaf area, and the number of leaves above the upmost ear is of great significance for improving maize yield.

Genome-wide association study (GWAS), a key technique for identifying variants associated with target traits by testing hundreds of thousands of genetic variants across many genomes (Uffelmann et al., 2021), have been successfully applied in plants (Spindel et al., 2016; Zegeye et al., 2014; Rice et al., 2020). Maize is regarded an ideal crop for GWAS due to its rich genetic diversity and rapid decay of linkage disequilibrium (LD) (Xiao et al., 2017; Jin et al., 2023; Sahito et al., 2024). A major locus for kernel oleic acid content was identified on chromosome 4 using whole genome scan association mapping in 553 elite maize inbred lines, and the putative candidate gene *fad2* was discovered (Beló et al., 2008). Following this discovery, GWAS has been widely employed to reveal the genetic basis of complex traits in maize, such as plant height, flowering time, disease resistance, drought tolerance, and grain yield (Ma et al., 2023; Zeng, 2024; Vanous et al., 2018; Mazaheri et al., 2019; Sahito et al., 2020). Samayoa et al. (2019) identified 39 SNPs that were significantly associated with resistance to *fumonisin* accumulation in maize kernels in 270 maize inbred lines. In the study of Jin et al. (2023), 19 significant SNPs were identified for chlorophyll content in 378 maize inbred lines.

A disadvantage of GWAS is the appearance of false positive associations due to individual relationships and population structure. Many statistical methods, such as the general linear model (GLM), the mixed linear model (MLM), FarmCPU, and BLINK, have been developed for GWAS to improve the statistical power and control of false positives. FarmCPU and BLINK are multi-locus test methods. FarmCPU effectively controls false positives and minimizes false negatives, allowing for rapid analysis of large populations with numerous markers (Liu et al., 2016). BLINK is an enhanced version of FarmCPU, which eliminates the assumption that genes controlling the target trait are evenly distributed across the genome (Wang and Zhang, 2021).

In recent years, numerous QTL, significant SNPs, and candidate genes conferring to leaf-related traits have been detected. Yang et al. (2016) conducted QTL mapping for leaf widths, leaf lengths, and leaf areas of eight consecutive leaves of maize below the tassel and grain yield using 253 RIL lines. A total of 171 and 159 putative QTLs were investigated using inclusive composite interval method and mixed-model-based composite interval mapping approach, respectively. Ma et al. (2018) identified 54 QTLs for leaf length, leaf width, leaf area, and leaf angle in an IBM Syn10 DH population. Guo et al. (2015) reported 46 QTLs related to the widths of leaves above the uppermost ear using four RIL populations. Tian et al. (2011) identified 36 and 34 QTLs for leaf length and leaf width by joint linkage mapping in the maize nest association mapping populations, respectively. Through the combined linkage mapping and association mapping, Dai et al. (2023) identified 290 QTLs and 165 SNPs controlling 25-leaf related traits, respectively. Five genetic loci were detected by both linkage mapping and associated mapping, and 77 genes were annotated. *Zm00001d026491* was a key candidate gene for leaf length. Using FarmCPU and BLINK methods, 19, 49, and 37 SNPs were identified for leaf length, leaf width, and leaf area, respectively (Zeng et al., 2025). A total of 57 candidate genes were retrieved. Li et al. (2018) reported a QTL *qLW10* associated with plant organ size in maize. The candidate gene, *ZmCSLD1*, encodes a cellulose synthase-like D protein that affects plant growth through cell division or expansion. The loss of *ZmCSLD1* function results in smaller leaf organs and reduced cell numbers. Although various studies have been reported for the genetic analysis of leaf-related traits, the genetic basis of leaf-related traits remains ambiguous.

Genomic selection (GS) was initially introduced by Meuwissen et al. (2001), who proposed that selection decisions could be made based on whole genome markers (de Koning, 2016). GS has made unprecedented progress in commercial breeding and gradually overtaken traditional marker-assisted selection (MAS) (Heffner et al., 2011). GS has been implemented widely in maize breeding (Romero Navarro et al., 2017; Riedelsheimer et al., 2012; Kadam et al., 2016; Liu et al., 2015, 2020). Zeng et al. (2025) conducted GS for leaf-related traits in a multi-parent DH population. The prediction accuracy for leaf length, leaf width, and leaf area was ranged from 0.30 to 0.43 based on the five-fold cross-validation method. Fan et al. (2024) conducted GS for maize flowering time-related traits. The prediction accuracy of days to tasseling, days to pollen-shedding, and days to silking was 0.47, 0.55, and 0.55, respectively. The GS prediction accuracy for ear shank length and ear shank node number was 0.39 and 0.37, respectively (Jiang et al., 2025). GS on grain yield, shelling percentage, kernel depth, tassel branch number, ear length, kernel number per ear, grain moisture at harvest, and 100 kernels weight were conducted based on the performance of the testcross (Sun et al., 2019). The prediction accuracy ranged from 18.65% to 66.2%. GS for husk tightness were conducted using six models, rrBLUP (Ridge Regression Best Linear Unbiased Prediction), BayesA, BayesB, BayesC, BL and BRR (Liu et al., 2023). The rrBLUB model with the highest GS prediction accuracy was the optimal prediction strategy. Kuki et al. (2020) conducted GS for Fusarium ear rot (FER) and starburst symptoms using genomic best linear unbiased prediction, Bayesian Lasso, and BayesC models in 320 tropical maize inbred lines. The prediction accuracy of all three models was very similar, ranging from 0.34 to 0.4 for FER and starburst, respectively. Song et al. (2024) found that incorporate genotype-by-environment (G×E) interaction in genomic selection can significantly improved the accuracy of genomic prediction. Although much progress has been made in GS for complex traits, there are few studies on GS for leaf-related traits.

In this study, 291 maize inbred lines were grown in four environments to evaluate the leaf length of the first leaf above the upmost ear (L1), the upmost ear leaf (L2), the first leaf below the upmost ear (L3), leaf width of L1, L2, and L3, leaf area of L2 (LAr2), and leaf number above the upmost ear (LNAE), and sequenced on the Illumina xplus platform for GWAS and GS. The main objectives of our study were to: (1) identify significant SNPs and candidate genes associated with leaf-related traits using two different GWAS models; (2) explore the potential of GS for the breeding of maize with improved leaf morphology; and (3) estimate the effect of training population size and marker density on GS prediction accuracy.

## 2 Materials and methods

### 2.1 Plant materials and field design

A natural population consisted of 291 diverse maize inbred lines was used for association mapping and GS. It was planted in four environments in China: (1) Qitai Experimental Station (N44° 5′, E89° 44′, PX23), Xinjiang, in May 2023; (2) Qitai Xidi Experimental Station (N44° 4′, E89° 44.4′, PD23), Xinjiang, in May 2023; (3) Ledong Experimental Station (N18° 27′, E108° 57′, PN24), Hainan, in October 2023; and (4) Erliugong Experimental Station (N44° 3′, E87° 10.2′, PQ24), Xinjiang, in May 2024. All the trials were conducted using a randomized complete block design with two replications per environment. Each plot consisted of two rows with five plants per row. The length of each row was 1.0m, and the distance between rows was 0.6m.

### 2.2 Phenotyping and data analysis

Leaf-related traits were evaluated 30 days after flowering. Five plants exhibiting consistent growth within each row were measured for the leaf length of L1 (LL1), L2 (LL2), and L3 (LL3), leaf width of L1 (LW1), L2 (LW2), and L3 (LW3), LAr2, and LNAE. Leaf length was measured from the leaf base to tip. Leaf length was measured at the widest point of the leaf. LNAE was the number of leaves above the upmost ear. LAr2 was calculated by leaf length of the upmost ear ×leaf width of the upmost ear ×0.75. The phenotypic data analysis was performed using R 4.3.2. Correlation analysis of leaf-related traits was conducted using the ‘corr.test’ function from the psych package. The “lmer” function in lme4 package (Bates et al., 2015) was employed to estimate the variance components, broad-sense heritability, and best linear unbiased prediction (BLUP) values across environments. The broad-sense heritability was estimated according to the method of Knapp et al. (1985) as follows:


H2= σg2σg2+σge2i+σe2ij

where $\sigma_{g}^{2}$ is the genotypic variance, $\sigma_{ge}^{2}$ is the genotype × environment interaction variance, $\sigma_{e}^{2}$ is the error variance, *i* and *j* represent the number of environments and the number of replications in each environment, respectively. All of the factors were considered as random effects when calculating heritability.

### 2.3 Genotyping

Young leaves of the 291 inbreds were harvested for DNA extraction. DNA extraction, library construction, and sequencing were performed at Beijing NovoGen Technology Co. Ltd (Beijing, China). DNA libraries were constructed with an insert size of 300 bp. The 150 bp paired-end reads were sequenced on the Illumina xplus platform with a sequencing depth of 5× coverage. Quality control of raw reads was conducted using Fastp V0.23.4 (Chen et al., 2018). Clean reads were aligned to the maize B73_RefGen_v5_ genome using BWA V0.1.17 (Li and Durbin, 2009). Duplicated sequences in the alignment results were removed using the dup option from the Picard V2.18.29 (Picard Tools. Broad Institute. http://broadinstitute.github.io/picard/) followed by variant calling using GATK V4.1.8 (Zhou et al., 2024). SNP filter was performed using the vcftools V0.1.16 (Danecek et al., 2011). Ultimately, 22,172,914 high-quality SNPs with heterozygosity rate less than 20%, minor allele frequency greater than 0.05, and missing rate less than 20% were obtained for GWAS and GS analysis.

### 2.4 Analyses of linkage disequilibrium and population structure

All the 22,172,914 SNPs were used for LD analysis. LD analysis and visualization were performed using PopLDdecay version 3.40 (Zhang et al., 2019). The average LD decay distance over all ten chromosomes with r^2^ = 0.1 was 33 kb (**Supplementary Figure 1**). Based on the LD decay distance, the number of SNP was reduced by LD pruning in a window size of 33 kb, and a step size of 10 by PLINK 1.9 (Purcell et al., 2007) Population structure analysis was performed using ADMIXTURE 1.3.0 software (Mussmann et al., 2020), with the K value set from 1 to 10. The K with the lowest cross-validation error was defined as the optimal K value.

### 2.5 Genome-wide association study

BLUP values across environments were utilized as the phenotypic data for GWAS. GWAS for leaf-related traits was conducted using the Bayesian-information and Linkage-disequilibrium Iteratively Nested Keyway (BLINK) and fixed and stochastic model cyclic probability unification (FarmCPU) models within the GAPIT package (Lipka et al., 2012) in R 4.3.2 (Null et al., 2011). The *p*-value threshold of 2.968 × 10^-6^, calculated by 1/the number of effective SNPs (1/336900), was used to identify significant SNPs. Manhattan plots and quantile-quantile (Q-Q) plots were performed using the R package ‘CMplot’ (https://github.com/YinLiLin/R-CMplot). Haplotype analysis was conducted using LDBlockShow (Dong et al., 2021).

### 2.6 Candidate gene analysis

According to the LD decay analysis, candidate genes were screened within 33 kb upstream and downstream of each significant SNPs based on the B73 RefGen_v5 reference genome. Candidate gene functions were annotated based on the National Center for Biotechnology Information [NCBI, https://www.ncbi.nlm.nih.gov/ (accessed on 5 August 2024)]. GO and KEGG pathway enrichment analyses were performed using agriGOV2 [http://systemsbiology.cau.edu.cn/agriGOv2/ (accessed on 22 October 2025)] and KOBAS 3.0 (http://bioinfo.org/kobas (accessed on 22 October 2025)), respectively. The protein interaction network analysis was conducted using STRING 12.0 [https://cn.string-db.org/ (accessed on 22 October 2025)]. The expression data of candidate genes was collected from MaizeGDB qTeller database [https://qteller.maizegdb.org/ (accessed on 1 September 2024)], and visualized using TBtools-II (Chen et al., 2023).

### 2.7 Genomic selection analysis

A total of 100,000 SNPs evenly distributed across 10 maize chromosomes were randomly selected for GS. GS was performed using the RR-BLUP model, which was conducted using the rrBLUP package in R 4.3.2. The prediction accuracy was calculated using a five-fold cross-validation method with 100 replications. To determine the optimal training population size, 10% to 90% (i.e. 10%, 20%, 30%, 40%, 50%, 60%, 70%, 80%, and 90%) of the 291 inbred lines were utilized as the training set, while the remaining lines were used as the validation set. To explore the impact of SNP numbers on prediction accuracy, 100 to 30,000 (i.e. 100, 300, 500, 1,000, 3,000, 5,000, 10,000, and 30,000) SNPs were selected for GS. In each marker density, SNPs were selected randomly 100 times to estimate the prediction accuracy.

## 3 Results

### 3.1 Phenotypic variation

Descriptive statistics of the eight leaf-related traits are presented in **Table 1**. Abundant phenotypic variations of leaf-related traits were observed in the GWAS panel. The mean value of leaf length ranged from 64.30 to 74.90cm for L1, from 64.27 to 76.62cm for L2, from 71.76 to 78.33cm for L3. The leaf width ranged from 7.55 to 10.50cm for L1, from 8.11 to 10.52cm for L2, from 7.68 to 10.40 for L3. The LAr2 ranged from 428.91 to 553.68 cm². The LNAE ranged from 5.87 to 6.61, The mean value of leaf-related traits varied in different environment. In combined analysis across environments, the overall mean of LL1, LL2, LL3. LW1, LW2, LW3, LAr2, and LNAE was 69.29cm, 72.06cm, 75.71cm, 9.11cm, 9.29cm, 9.07cm, 507.66 cm², and 6.16, respectively. L3 showed the longest leaf length and L2 showed the widest leaf width. The coefficients of variation (CV) for the eight leaf-related traits ranged from 4.70% to 19.43%. All the leaf-related traits were approximated as a normal distribution.

**Table 1:** Statistical analysis of leaf-related traits in each environment and the combined-environment.

Trait^a^	Environment^b^	Mean ± SD	Min	Max	Skewness	Kurtosis	CV (%)^c^
LL1 (cm)	PX23	74.90 ± 6.50	48.90	89.70	-0.57	0.60	8.68
	PD23	64.30 ± 4.65	51.10	87.50	0.63	0.81	7.22
	PN24	70.75 ± 7.55	49.80	91.70	0.09	-0.19	10.67
	PQ24	67.24 ± 5.90	46.97	85.40	-0.18	0.45	8.78
	Combined	69.29 ± 3.64	58.09	78.26	-0.23	0.09	5.25
LL2 (cm)	PX23	75.27 ± 8.05	53.10	98.20	-0.11	-0.08	10.69
	PD23	71.91 ± 5.38	52.60	89.30	0.04	0.57	7.49
	PN24	76.62 ± 5.86	59.00	91.70	-0.25	-0.12	7.64
	PQ24	64.27 ± 5.98	46.20	89.70	0.56	0.89	9.30
	Combined	72.06 ± 3.39	62.35	80.14	-0.25	-0.04	4.70
LL3 (cm)	PX23	75.98 ± 8.68	51.70	98.00	-0.06	-0.23	11.43
	PD23	71.76 ± 5.45	52.50	88.50	0.01	0.52	7.59
	PN24	76.63 ± 6.02	59.20	92.80	-0.27	0.12	7.86
	PQ24	78.33 ± 9.52	46.90	111.20	0.43	0.76	12.15
	Combined	75.71 ± 4.61	61.12	87.20	-0.26	0.34	6.09
LW1 (cm)	PX23	9.63 ± 1.33	5.70	14.40	0.29	0.32	13.82
	PD23	7.55 ± 1.13	5.43	13.00	0.27	0.98	14.91
	PN24	10.50 ± 1.19	6.73	14.60	0.26	0.02	11.38
	PQ24	8.76 ± 1.08	6.00	12.30	0.22	-0.20	12.31
	Combined	9.11 ± 0.65	7.06	11.06	0.15	0.23	7.13
LW2 (cm)	PX23	9.58 ± 1.35	5.90	14.10	0.22	0.11	14.08
	PD23	8.11 ± 0.93	5.40	12.20	0.68	0.92	11.49
	PN24	10.52 ± 1.21	6.60	14.70	0.32	0.22	11.48
	PQ24	8.90 ± 1.06	6.40	12.00	0.20	-0.25	11.87
	Combined	9.29 ± 0.65	7.26	11.29	0.13	0.29	7.02
LW3 (cm)	PX23	9.31 ± 1.35	5.40	13.40	0.17	-0.04	14.53
	PD23	7.68 ± 1.13	5.20	12.90	0.85	0.95	14.68
	PN24	10.40 ± 1.23	6.33	14.60	0.30	0.20	11.79
	PQ24	8.84 ± 1.07	6.30	12.10	0.12	-0.30	12.09
	Combined	9.07 ± 0.69	7.16	11.31	0.21	0.26	7.61
LAr2 (cm^2^)	PX23	552.50 ± 107.36	277.70	1023.70	0.36	0.57	19.43
	PD23	428.91 ± 60.28	268.70	669.40	0.76	0.96	14.05
	PN24	493.77 ± 91.28	279.50	766.80	0.37	-0.18	18.49
	PQ24	553.68 ± 93.54	304.40	915.50	0.51	0.32	16.89
	Combined	507.66 ± 54.13	349.18	651.10	0.05	0.02	10.66
LNAE	PX23	6.61 ± 0.80	5.00	9.00	0.39	-0.10	12.10
	PD23	6.1 ± 0.53	4.00	8.00	-0.03	0.16	8.62
	PN24	6.04 ± 0.59	5.00	8.00	0.24	-0.26	9.73
	PQ24	5.87 ± 0.71	4.00	8.00	0.28	-0.05	12.19
	Combined	6.16 ± 0.46	5.16	7.53	0.28	-0.27	7.43

^a^LL1, length of the first leaf above the uppermost ear; LL2, length of the uppermost ear leaf; LL3, length of the first leaf below the uppermost ear; LW1, width of the first leaf above the uppermost ear; LW2, width of the uppermost ear leaf; LW3, width of the first leaf below the uppermost ear; LAr2, leaf area of the uppermost ear; LNAE, total number of leaves above the uppermost ear. ^b^PX23, Qitai Experimental Station in May 2023; PD23, Qitai Xidi Experimental Station in May 2023; PN24, Ledong Experimental Station in October 2023; PQ24, Erliugong Experimental Station in May 2024; Combined, the combined-environment. ^c^CV, coefficient of variation.

**Figure 1:**
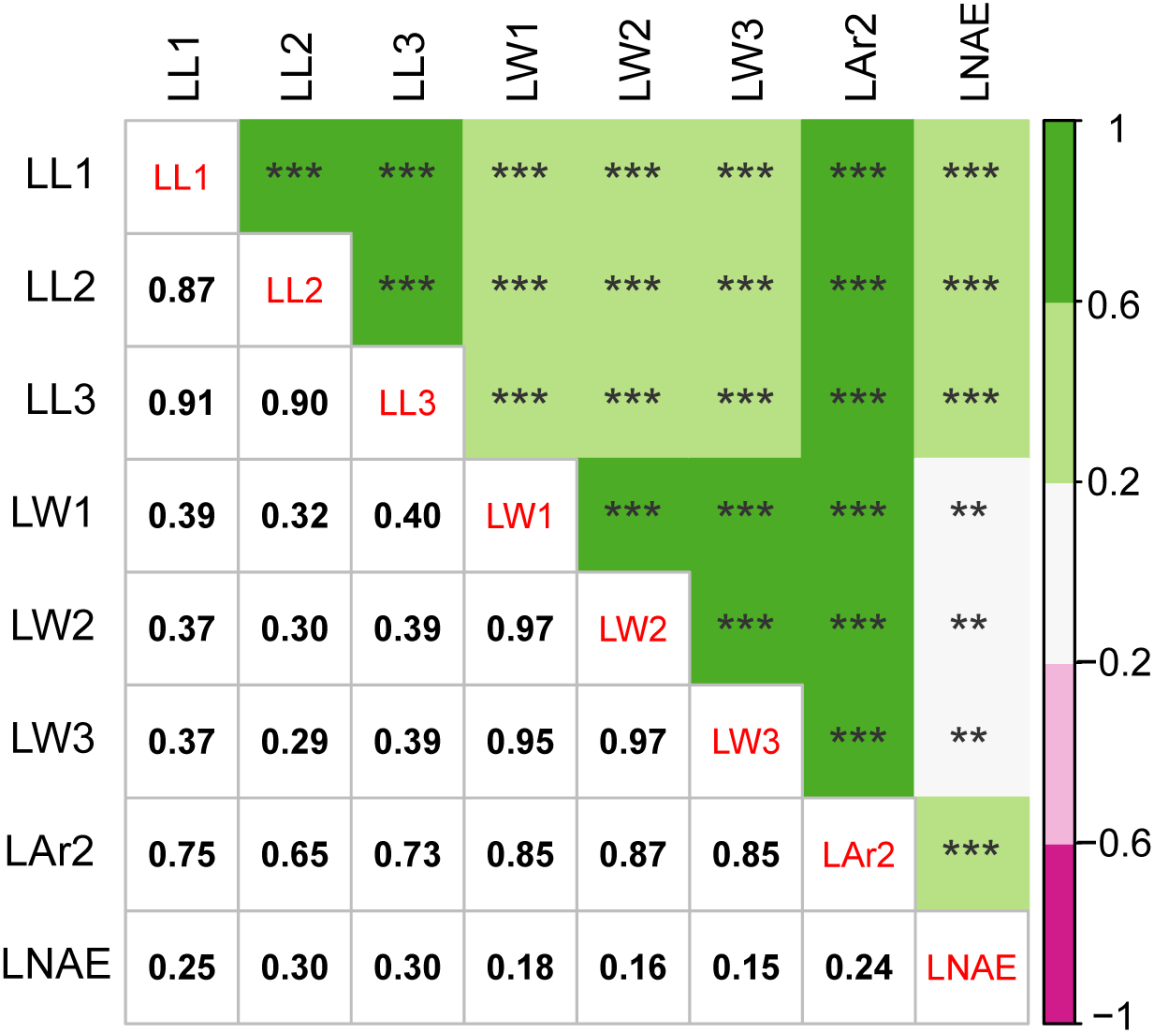
Correlation analysis for leaf-related traits in 291 maize inbred lines. The value below the diagonal is the Pearson correlation coefficient (r) values between each pair of traits. ** significant at the 0.01 level; *** significant at the 0.001 level. LL1, length of the first leaf above the uppermost ear; LL2, length of the uppermost ear leaf; LL3, length of the first leaf below the uppermost ear; LW1, width of the first leaf above the uppermost ear; LW2, width of the uppermost ear leaf; LW3, width of the first leaf below the uppermost ear; LAr2, leaf area of the uppermost ear; LNAE, total number of leaves above the uppermost ear.

Significant (*P* < 0.001) genotypic variance and genotype-environment interaction variance were found for leaf-related traits (**Table 2**). The broad-sense heritability was 77.93%, 79.05%, 82.51%, 80.12%, 80.42%, 81.91%, 81.20%, and 87.54% for LL1, LL2, LL3, LW1, LW2, LW3, LAr2, and LNAE, respectively. Correlation analysis revealed significant (*P* < 0.01) positive correlation between leaf-related traits (**Figure 1**). The Pearson’s correlation coefficient ranged from 0.15 to 0.97. Higher correlations existed between the same traits of leaves at different positions than that between different traits, indicating that the same traits of leaves in different positions have higher genetic similarities.

**Table 2:** Variance components and broad-sense heritability (*H^2^*) of leaf-related traits in 291 maize inbred lines.

Traits^a^	Variance Components^b^			*H*^2^ (%)^c^
	$\sigma_{g}^{2}$	${\text{σ}}_{ge}^{2}$	$\sigma_{e}^{2}$	
LL1	17.29^***^	16.29^***^	6.59^***^	77.93
LL2	14.79^***^	5.57^***^	20.22^***^	79.05
LL3	25.92^***^	11.42^***^	21.10^***^	82.51
LW1	0.54^***^	0.20^***^	0.66^***^	80.12
LW2	0.54^***^	0.26^***^	0.52^***^	80.42
LW3	0.59^***^	0.21^***^	0.63^***^	81.91
LAr2	3738.98^***^	2355.25^***^	2213.72^***^	81.20
LNAE	0.24^***^	0.07^***^	0.13^***^	87.54

^a^LL1, length of the first leaf above the uppermost ear; LL2, length of the uppermost ear leaf; LL3, length of the first leaf below the uppermost ear; LW1, width of the first leaf above the uppermost ear; LW2, width of the uppermost ear leaf; LW3, width of the first leaf below the uppermost ear; LAr2, leaf area of the uppermost ear; LNAE, total number of leaves above the uppermost ear. ^b^ $\sigma_{\text{g}}^{2}$, genotypic variance; ${\text{σ}}_{\text{ge}}^{2}$, genotype × environment interaction variance; $\sigma_{e}^{2}$, error variance. *** significant at the 0.001 level. ^c^broad-sense heritability.

### 3.2 SNP information and population structure analysis

**Figure 2:**
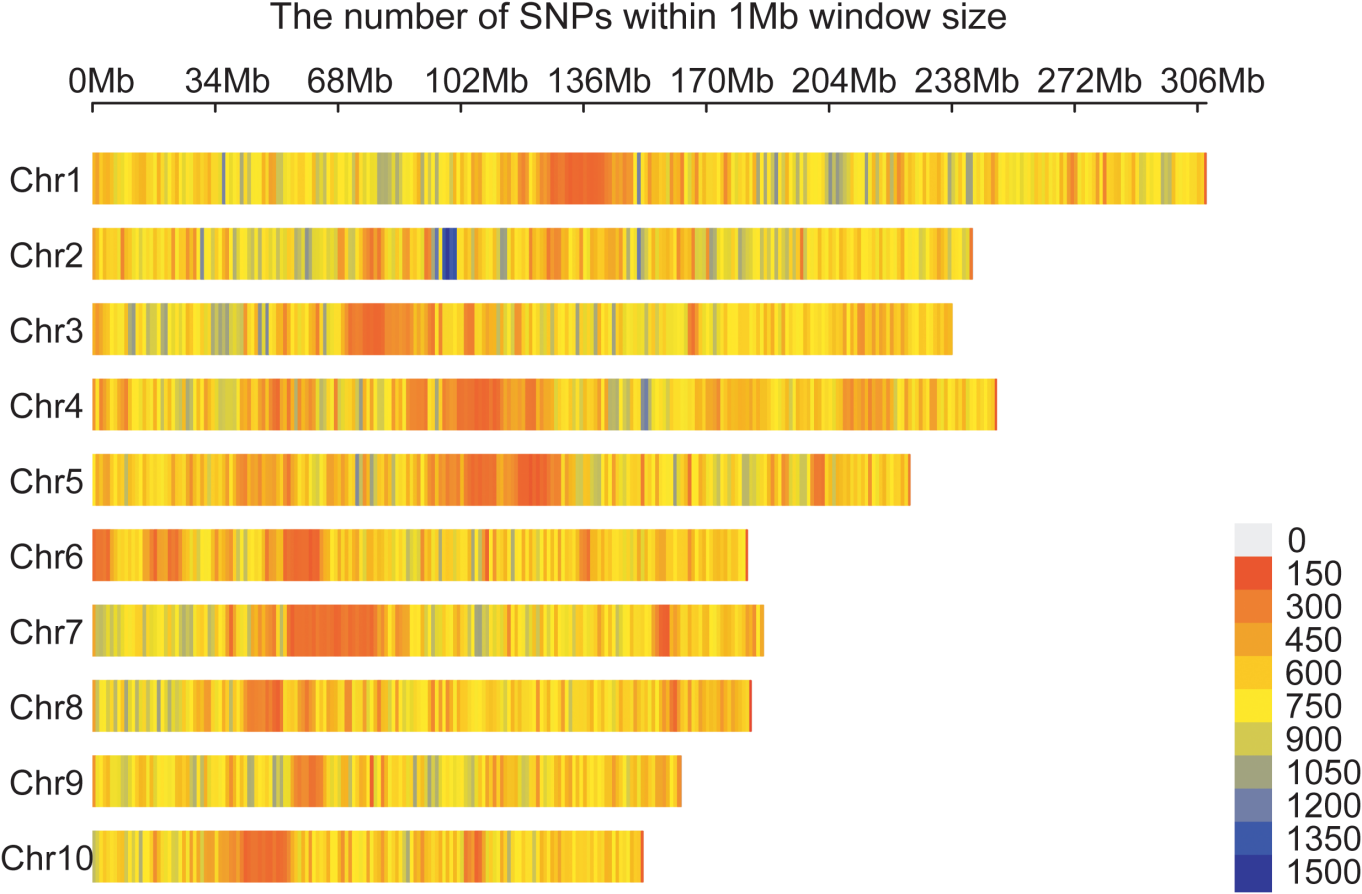
Distribution of 1,343,525 SNPs across 10 maize chromosomes. A density distribution map of SNPs across the 10 chromosomes in 1 Mb genomic interval.

A total of 1,343,525 high-quality SNPs evenly distributed across 10 maize chromosomes were retained and used for GWAS and GS (**Figure 2**). The number of SNPs on each chromosome range from 90471 on chromosome 10 to 206525 on chromosome 1, with an average of 13,435 SNPs.

**Figure 3:**
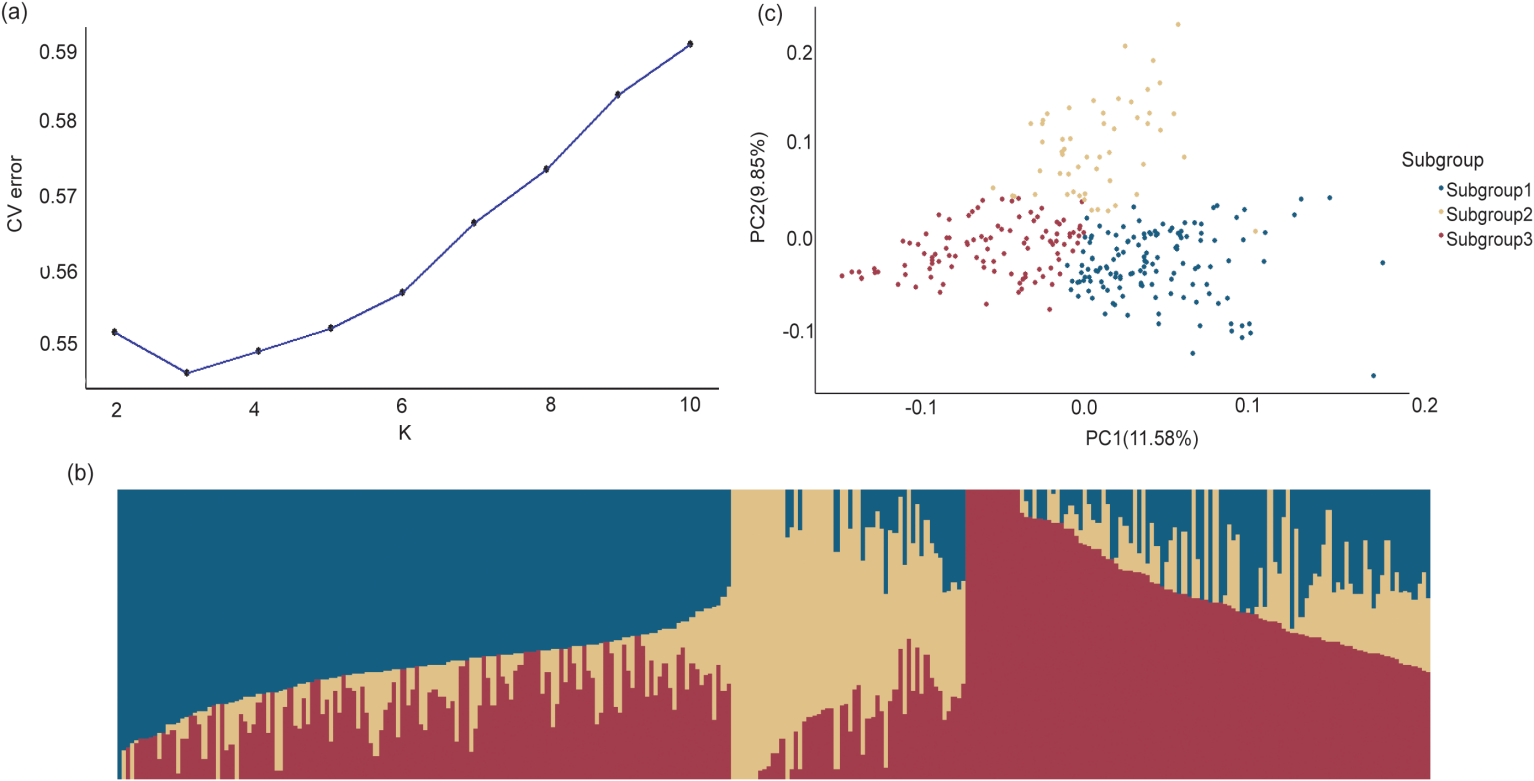
Population structure analysis of the GWAS panel based on 1,343,525 SNPs. **(a)** The plot of delta K of 291 maize inbred lines. **(b)** The estimate probability membership for each line at K=3. **(c)** The principal component analysis plots. Different colors represent different subgroups identified by STRUCTURE analysis.

The results of population structure are presented in **Figure 3**. When K=3, the cross-validation error was the lowest compared to other K values, indicating that the association panel could be divided into three subgroups (**Figure 3A**). Subgroup 1, Subgroup 2, and Subgroup 3 contained 135, 53, and 103 inbred lines, respectively (**Figure 3B**), which was consistent with the results of principal component analysis (PCA) (**Figure 3C**).

### 3.3 Genome-wide association analysis of leaf-related traits

**Figure 4:**
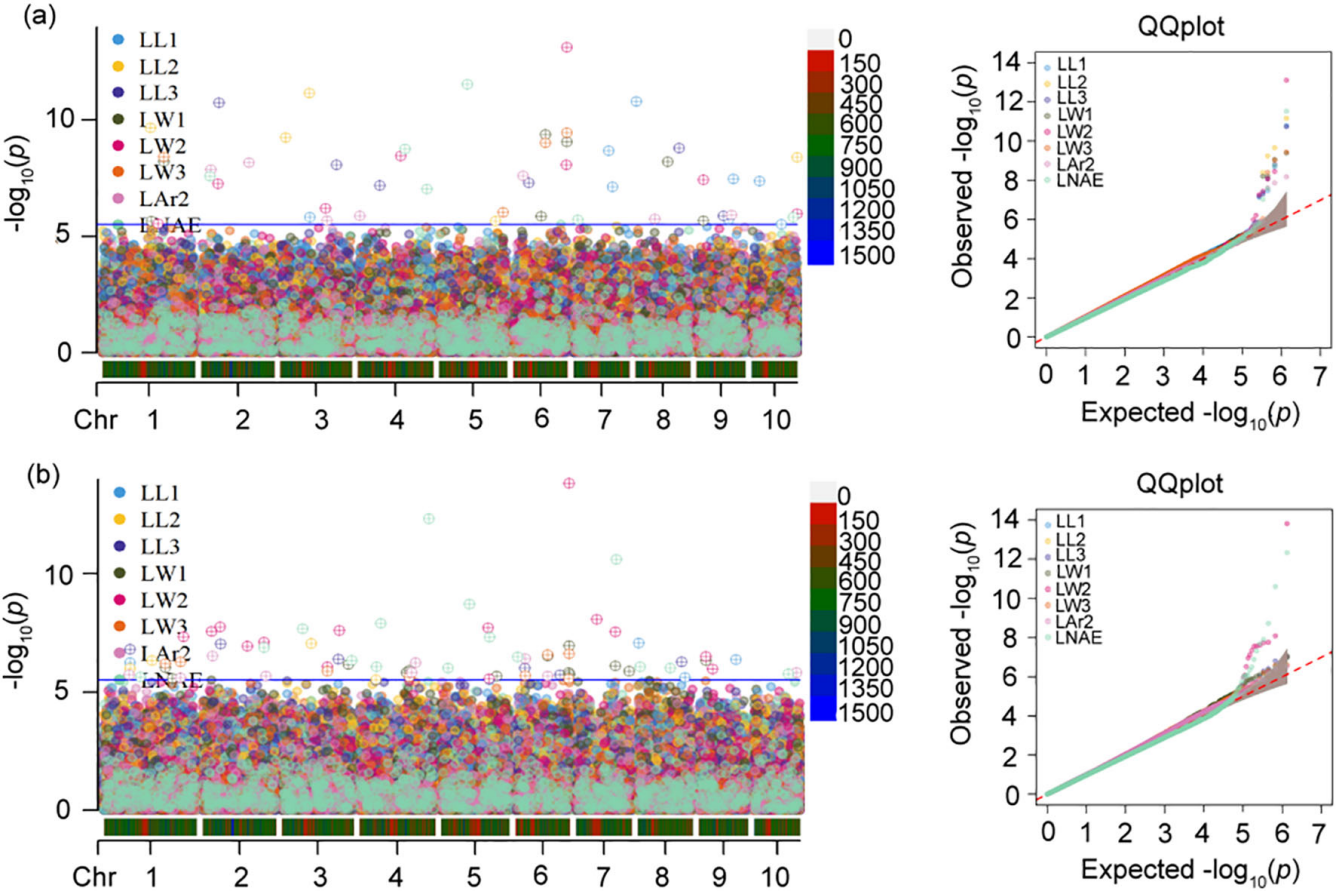
Manhattan and quantile–quantile (q–q) plots of genome-wide association study (GWAS) for leaf-related traits using **(a)** BLINK model and **(b)** FarmCPU model. In Manhattan plot, the solid blue horizontal line indicates the *P*-value threshold of 2.97×10^-6^. LL1, length of the first leaf above the uppermost ear; LL2, length of the uppermost ear leaf; LL3, length of the first leaf below the uppermost ear; LW1, width of the first leaf above the uppermost ear; LW2, width of the uppermost ear leaf; LW3, width of the first leaf below the uppermost ear; LAr2, leaf area of the uppermost ear; LNAE, total number of leaves above the uppermost ear.

The GWAS results of leaf-related traits are presented in **Figure 4** and **Supplementary Table 1**. At the *p*-value threshold of 2.968 × 10^-6^, 51 and 73 SNPs controlling leaf-related traits were identified by BLINK and FarmCPU, respectively. According to quantile-quantile (Q-Q) plots, population structure was well controlled by both BLINK and FarmCPU models.

For LL1, eight SNPs located on chromosomes 3, 7, 8, 9, and 10 were identified by BLINK. The phenotypic variance explained (PVE) by each SNP ranged from 4.82% to 16.10%. The most significant SNP 8_2077268 exhibited the lowest *p*-value of 1.61×10^-11^, accounting for 16.10% of the phenotypic variation. Four SNPs associated with LL1 were identified on chromosomes 1, 8, and 9 by FarmCPU. The PVE ranged from 11.80% to 19.20%. The most significant SNP 8_2077268 showed the lowest *p*-value of 8.43×10^-8^, accounting for 14.90% of phenotypic variation. Two SNPs 8_2077268 and 9_122936513 were detected by both models.

For LL2, five SNPs located on chromosomes 1, 3, 5, and 10 were identified by BLINK, and explained 8.00% to 20.70% of phenotypic variation. The most significant SNP 3_95871968 had the lowest *p*-value of 6.89×10^-12^, explaining 14.10% of the phenotypic variation. Four SNPs related to LL2 were detected on chromosomes 1, 3, and 4 by FarmCPU, with the PVE ranging from 11.70% to 16.90%. The most significant SNP 3_95871968 showed the lowest *p*-value of 8.62×10^-8^, accounting for 16.90% of phenotypic variation. Two SNPs, 1_159903081 and 3_95871968, were detected by both models.

For LL3, six SNPs located on chromosomes 2, 3, 4, 6, 8, and 9 were identified by BLINK, and explained 9.37% to 15.30% of phenotypic variation. The most significant SNP 2_58557744 showed the lowest *p*-value of 1.85×10^-11^, explaining 12.70% of the phenotypic variation. Six SNPs associated with LL3 were detected on chromosomes 1, 2, 3, 6, and 8 by FarmCPU, with the PVE ranging from 6.99% to 15.80%. The most significant SNP 2_58557744 had the lowest *p*-value of 9.37×10^–8^ and a PVE of 8.31%. Three SNPs 2_58557744, 3_187354520, and 8_144381051 were identified by both models.

For LW1, seven SNPs located on chromosomes 1, 6, 8, and 9 were identified by BLINK, and explained 4.10% to 16.90% of phenotypic variation. The most significant SNP 6_107526600 showed the lowest *p*-value of 4.11×10^-10^, explaining 16.90% of the phenotypic variation. Thirteen SNPs associated with LW1 were detected on chromosomes 1, 3, 4, 5, 6, 7, and 9 by FarmCPU, with the PVE ranging from 0.01% to 8.50%. The most significant SNP 6_178934629 had the lowest *p*-value of 1.09×10^–7^ and a PVE of 5.36%. Four SNPs 1_204491759, 6_107526600, 6_178934629, and 9_21919877 were identified by both models.

For LW2, eight SNPs located on chromosomes 1, 2, 3, 4, 6, 9, and 10 were identified by BLINK, and explained 4.94% to 17.00% of phenotypic variation. The most significant SNP 6_178934629 exhibited the lowest *p*-value of 7.67×10^–14^ and explaining 4.94% of the phenotypic variance. Fourteen SNPs associated with LW2 were detected on all ten chromosomes except for chromosomes 4, 8, and 10 by FarmCPU, with the PVE ranging from 1.31% to 15.30%. The most significant SNP 6_178934629 had the lowest *p*-value of 1.51×10^–14^ and a PVE of 3.81%. Three SNPs 3_150800464, 6_178934629, and 9_21919877 were identified by both models.

For LW3, four SNPs located on chromosomes 1, 5, and 6 were identified by BLINK, and explained 4.56% to 17.20% of phenotypic variation. The most significant SNP 6_178934629 exhibited the lowest *p*-value of 3.54×10^–10^ and explaining 12.30% of the phenotypic variance. Eight SNPs associated with LW3 were detected on chromosomes 1, 3, 4, and 6 by FarmCPU, with the PVE ranging from 2.56% to 9.04%. The most significant SNP 6_178934629 had the lowest *p*-value of 2.42×10^–7^ and a PVE of 7.24%. Three SNPs 1_204491759, 6_107526600, and 6_178934629 were identified by both models.

For LAr2, seven SNPs located on chromosomes 2, 3, 4, 6, 8, and 9 were identified by BLINK, and explained 2.86% to 21.80% of phenotypic variation. The most significant SNP 2_158371143 exhibited the lowest *p*-value of 6.60×10^–9^ and explaining 4.83% of the phenotypic variance. Nine SNPs associated with LAr2 were detected on chromosomes 1, 2, 4, 6, and 10 by FarmCPU, with the PVE ranging from 0.70% to 15.10%. The most significant SNP 2_31225540 had the lowest *p*-value of 3.00×10^–7^ and a PVE of 13.90%. Two SNPs 2_31225540 and 6_31979816 were identified by both models.

For LNAE, six SNPs located on chromosomes 2, 4, 5, 7, and 10 were identified by BLINK, and explained 5.12% to 25.01% of phenotypic variation. The most significant SNP 5_93495559 exhibited the lowest *p*-value of 2.97×10^–12^ and explaining 13.40% of the phenotypic variance. Fifteen SNPs associated with LNAE were detected on all ten chromosomes except for chromosome 9 by FarmCPU, with the PVE ranging from 1.21% to 14.60%. The most significant SNP 4_230408762 had the lowest *p*-value of 4.68×10^–13^ and a PVE of 14.60%. Two SNPs 4_230408762 and 5_93495559 were identified by both models.

**Figure 5:**
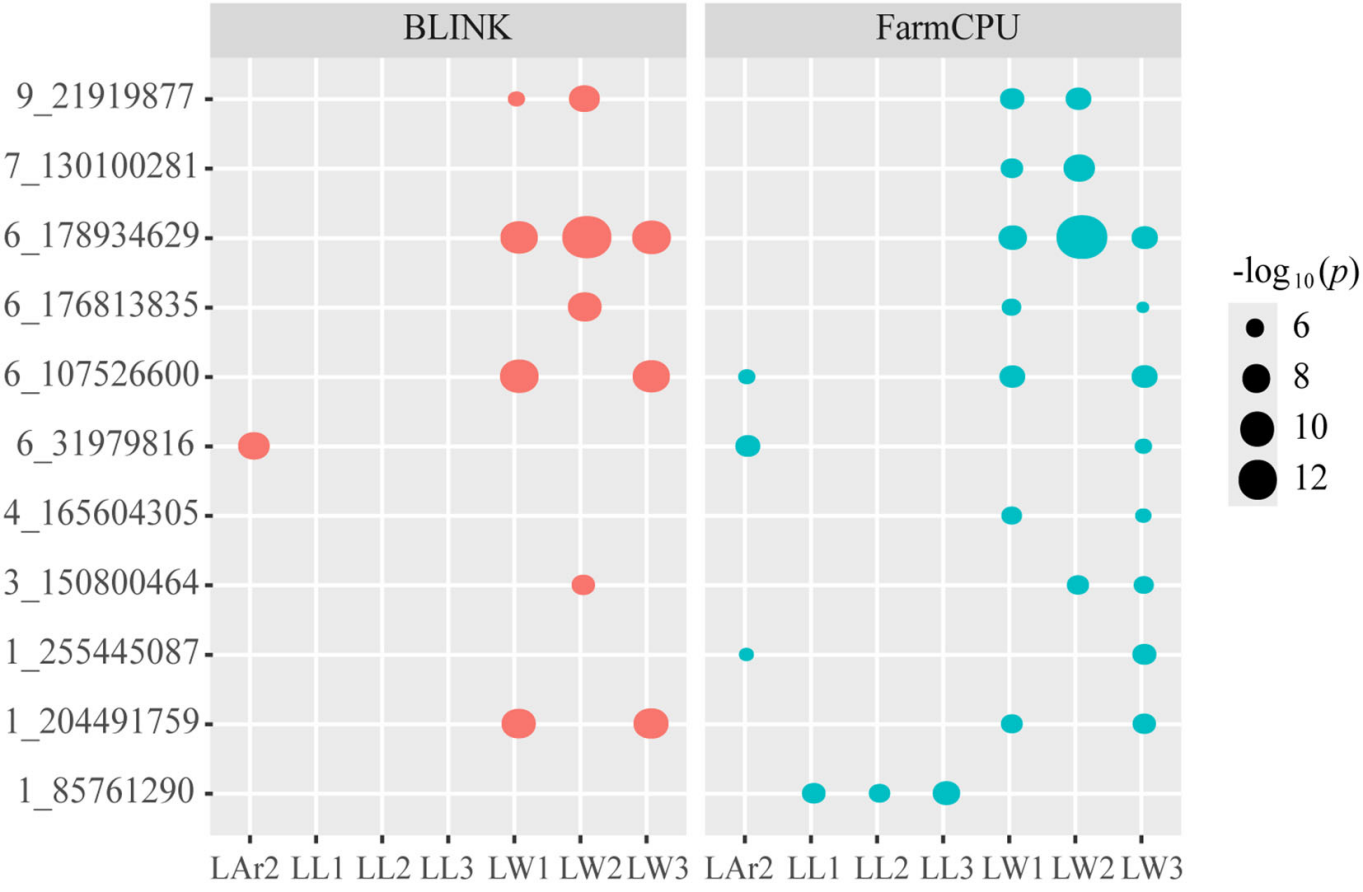
Bubble plot analysis of the 11 pleiotropic SNPs controlling leaf-related traits. Red represent BLINK model and blue represent FarmCPU model. The x-axis displays eight leaf-related traits, and the y-axis shows 11 pleiotropic SNPs. The size of bubbles indicates the level of significance, with larger bubbles representing higher significance. LL1, length of the first leaf above the uppermost ear; LL2, length of the uppermost ear leaf; LL3, length of the first leaf below the uppermost ear; LW1, width of the first leaf above the uppermost ear; LW2, width of the uppermost ear leaf; LW3, width of the first leaf below the uppermost ear; LAr2, leaf area of the uppermost ear.

Eleven pleiotropic SNPs, 1_85761290, 1_204491759, 1_255445087, 3_150800464, 4_165604305, 6_107526600, 6_178934629, 6_31979816, 6_176813835, 7_130100281, and 9_21919877, controlling leaf-related traits were identified (**Table 3**, **Figure 5**). SNP 1_85761290 located at 1.05 was identified for the three leaf length traits LL1, LL2, and LL3. SNP 6_176813835 located at bin 6.07 and SNP 6_178934629 located at bin 6.08 were detected for three leaf width traits LW1, LW2, and LW3. SNP 6_107526600 located at bin 6.03 was identified for LW1, LW3, and LAr2. SNP 1_204491759 located at bin 1.07 and SNP 4_165604305 located at bin 4.06 were identified for LW1 and LW3. SNP 7_130100281 located at bin 7.02 and SNP 9_21919877 located at bin 9.02 were identified for LW1 and LW2. SNP 3_150800464 located at bin 3.05 was identified for LW2 and LW3. SNP 1_255445087 located at bin 1.08 and SNP 6_31979816 located at bin 6.01 were identified for LW3 and LAr2.

**Table 3:** Candidate genes for 11 pleiotropic SNPs controlling leaf-related traits.

SNP^a^	Trait^b^	Candidate gene^c^	Gene annotation^d^
1_85761290	LL1, LL2, LL3	\	\
1_204491759	LW1, LW3	Zm00001eb038260	Uncharacterized protein
		Zm00001eb038270	Patatin-like protein 2-like
1_255445087	LW3, LAr2	Zm00001eb050120	Os03g0604500-like protein
		Zm00001eb050130	Serine-threonine protein kinase plant-type
		Zm00001eb050140	subtilisin-like protease
3_150800464	LW2, LW3	\	\
4_165604305	LW1, LW3	Zm00001eb188530	Carbamoyl-phosphate synthase small chain, chloroplastic
6_107526600	LW1, LW3, LAr2	Zm00001eb275550	STRUBBELIG family receptor protein kinase
		Zm00001eb275560	Nop domain-containing protein
		Zm00001eb275570	Nudix hydrolase 2-like
6_178934629	LW1, LW2, LW3	Zm00001eb297330	Plant cysteine oxidase 2
6_176813835	LW1, LW2, LW3	Zm00001eb296310	putative timeless C-term domain and Homeodomain protein
		Zm00001eb296320	DUF3143 family protein
		Zm00001eb296330	Uncharacterized protein
		Zm00001eb296340	G-box binding factor 1
		Zm00001eb296350	glucose-6-phosphate 1-epimerase
		Zm00001eb296360	Uncharacterized protein
		Zm00001eb296370	Uncharacterized protein
6_31979816	LW3, LAr2	\	\
7_130100281	LW1, LW2	Zm00001eb314670	Beta-1,3-galactosyltransferase 7
9_21919877	LW1, LW2	Zm00001eb377180	AP2-like ethylene-responsive transcription factor SMOS1

^a^SNP names, chromosome position, 1_85761290 refers that the SNP is located on chromosome 1 with the physical position of 85761290 bp. ^b^LL1, length of the first leaf above the uppermost ear; LL2, length of the uppermost ear leaf; LL3, length of the first leaf below the uppermost ear; LW1, width of the first leaf above the uppermost ear; LW2, width of the uppermost ear leaf; LW3, width of the first leaf below the uppermost ear; LAr2, leaf area of the uppermost ear. ^c^Candidate genes identified based on B73 RefGen_v5. ^d^Functional annotation information for gene in NCBI.

**Figure 6:**
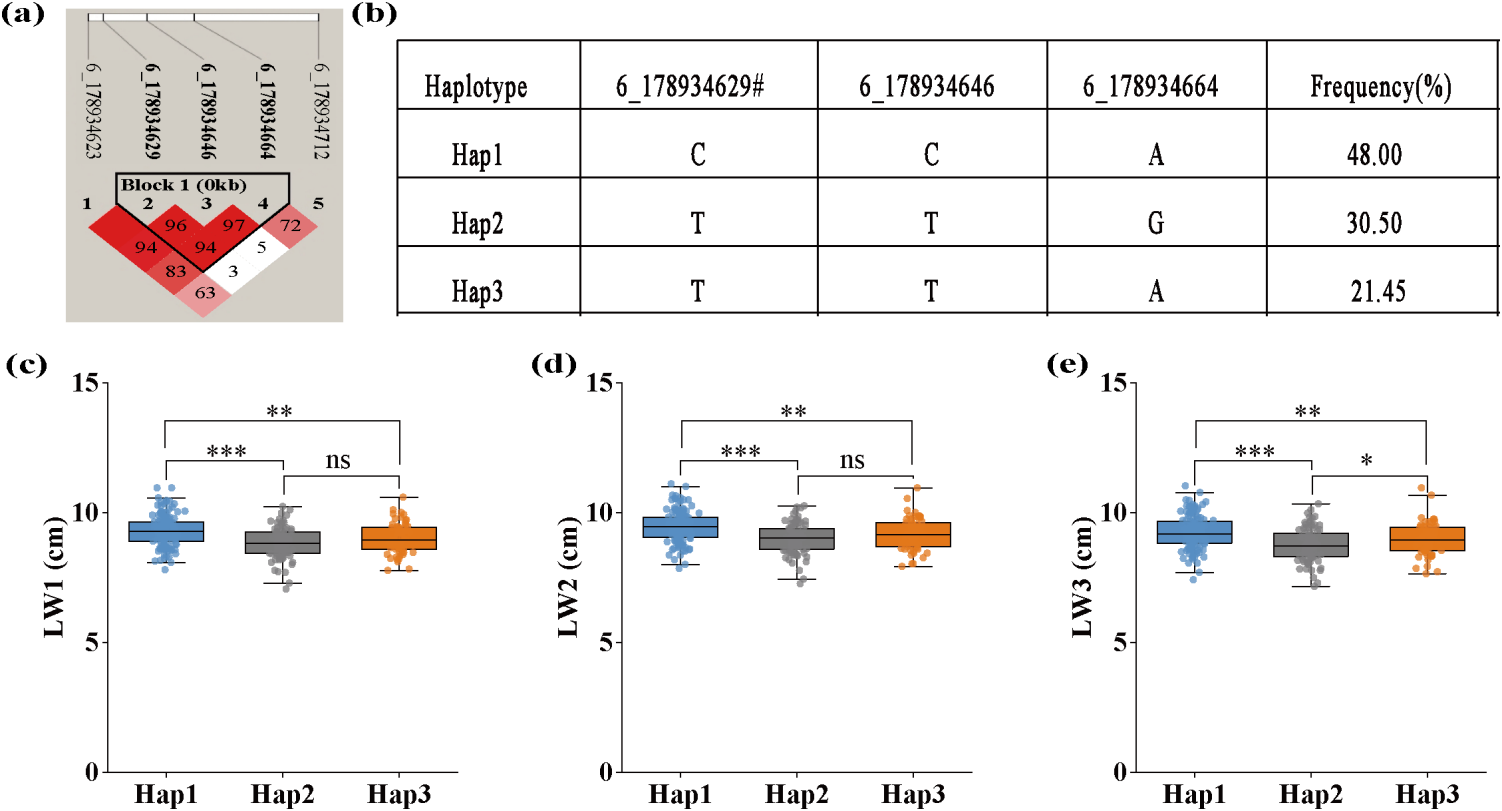
Haplotype analysis of pleiotropic SNP 6_178934629 identified for LW1, LW2, and LW3 using both FarmCPU and BLINK models. **(a)** The linkage disequilibrium (LD) heatmap of SNPs surrounding SNP 6_178934629, where color intensity (as indicated by the R^2^ color scale) and numerical values within color blocks represent the LD value (r^2^) between marker pairs. **(b)** Three haplotypes of SNP 6_178934629 discovered in 291 maize inbred lines. **(c)** The effect of different haplotypes on LW1, width of the first leaf above the uppermost ear. **(d)** The effect of different haplotypes on LW2, width of the uppermost ear leaf. **(e)** The effects of different haplotypes on LW3. *significant at the 0.05 level; **significant at the 0.01 level; ***significant at the 0.001 level.

SNP 6_178934629 was not only co-localized with LW1, LW2, and LW3, but also identified by both models for each trait. Haplotype analysis of SNP 6_178934629 was performed and the result is shown in **Figure 6**. SNP 6_178934629 was located in the same haplotype block with SNP 6_178934646 and 6_178934664 (**Figure 6a**). Three haplotypes “CCA”, “TTG”, and “TTA”, were detected in our GWAS panel (**Figure 6b**). The Hap1 (“CCA”) showed the widest leaf with of L1 (**Figure 6c**), L2 (**Figure 6d**), and L3 (**Figure 6e**), with a frequency of 48.00%. The Hap2 (“TTG”) showed the narrowest leaf with of L1, L2, and L3, with a frequency of 30.55%. According to the breeding objectives, suitable haplotypes could be selected for molecular breeding.

### 3.4 Functional annotation of candidate genes

Based on the B73 RefGen_v5 reference genome, 122 candidate genes were identified within 33 kb upstream and downstream of each SNP significantly associated with all eight leaf-related traits, and 103 genes were annotated with known functions (**Supplementary Table 2**). The number of candidate genes identified for LL1, LL2, LL3, LW1, LW2, LW3, LAr2, and LNAE was 9, 6, 5, 28, 34, 17, 26, and 27, respectively. A total of 19 candidate genes were identified for the 11 pleiotropic SNPs, including 15 candidate genes with known functions (**Table 3**).

A total of 95 candidate genes were annotated with 181 Gene Ontology (GO) terms, categorized into 93 for biological processes (BP), 41 for cellular components (CC), and 47 for molecular functions (MF) (**Supplementary Table 3**). GO analysis showed no significant enrichment in any particular GO term (false discovery rate < 0.05), suggesting that leaf-related traits were influenced by multiple biological mechanisms, rather than a few highly integrated mechanisms. The top ten GO terms of BP, CC, and MF are presented in **Supplementary Figure 2**. The Four terms include plastid thylakoid (GO:0031976), organelle subcompartment (GO:0031984), thylakoid (GO:0009579), chromosome (GO:0005694), and transmembrane transport (GO:0055085) with *p*-values < 0.05, may be involved in maize leaf development. Additionally, 16 candidate genes were mapped to 26 KEGG pathways (**Supplementary Table 4**). KEGG analysis also showed no significant enrichment. This may due to the limited number of genes annotated into KEGG pathways. Protein-protein interaction (PPI) analysis identified 12 genes forming three distinct interaction clusters (**Supplementary Figure 3**, **Supplementary Table 5**). Cluster 1 comprises six proteins, including factors associated with high chlorophyll fluorescence, an Alba DNA/RNA-binding protein, a thioredoxin superfamily protein, and ribosomal proteins. Cluster 2 contains four proteins, such as breast cancer susceptibility type 2 homolog B, replication factor C subunit 2, a putative Timeless C-terminal domain-containing protein, and a homeodomain protein. Cluster 3 consists of two proteins: a putative polyphenol oxidase and a chromatin remodeling factor 24.

**Figure 7:**
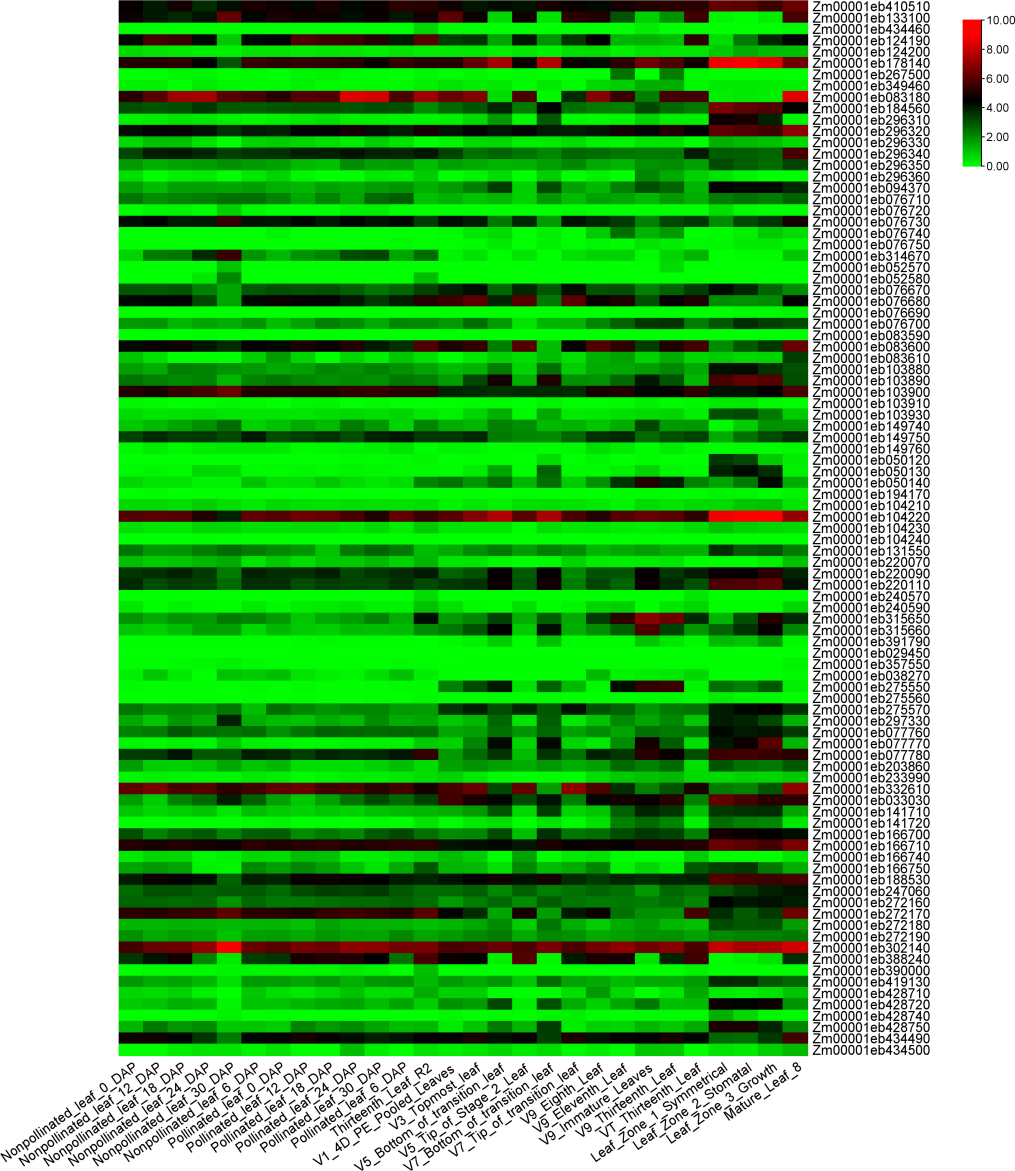
The expression heatmap of the 93 candidate genes identified for leaf-related traits in 28 leaf-related tissues. The figure shows the expression values of candidate genes, which are the log2(n+1) conversion values of FPKM count.

To investigate the expression patterns of the 122 candidate genes, the expression levels of 28 different leaf tissues of B73 was downloaded and analyzed. Twenty-nine genes showed very low or no expression in the 28 tissues. The expression patterns of the rest 93 genes were shown in **Figure 7**. Two genes, *Zm0001eb104220* and *Zm0001eb302140* exhibited medium to high expression levels in all the 28 leaf tissues. Gene *Zm0001eb083180* showed high expression levels in most leaf tissues, especially in the basal leaf meristematic tissues from the three youngest leaves of maize plants at the 12-leaf stage (Kakumanu et al., 2012), and mature leaf 8 (43 days old) (Walley et al., 2016). *Zm0001eb178140* showed relatively high expression level in 3 zones from leaf 8 (30 days old) (Walley et al., 2016).

### 3.5 Genomic selection prediction accuracy estimated using different training population sizes and marker densities

The GS prediction accuracies estimated through 5-fold cross-validation were 0.25, 0.22, 0.25, 0.41, 0.44, 0.44, 0.41, and 0.31 for LL1, LL2, LL3, LW1, LW2, LW3, LAr2, and LNAE, respectively. LW2 exhibited the highest prediction accuracy, while LL2 showed the lowest prediction accuracy (**Supplementary Table 6**).

**Figure 8:**
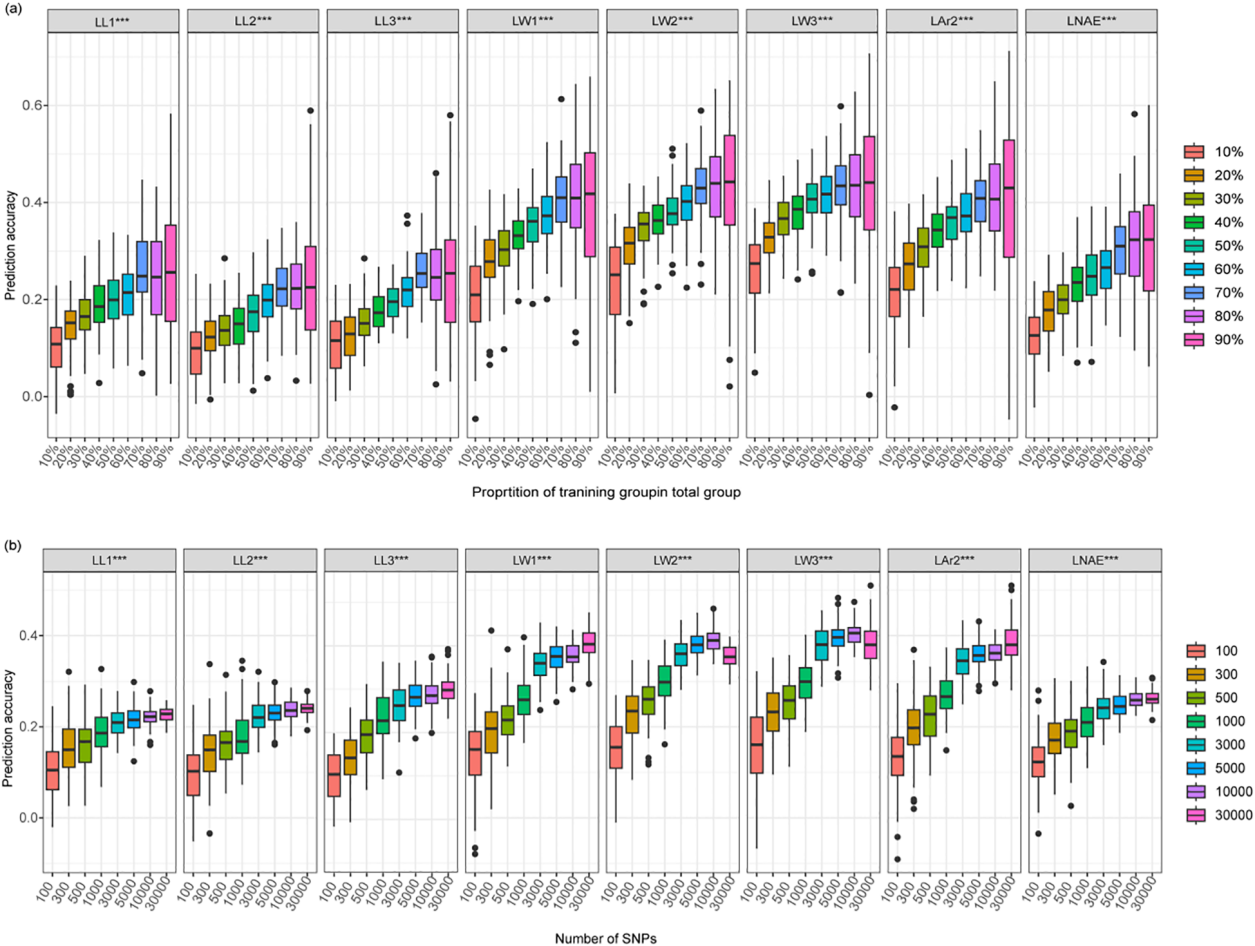
Genomic prediction accuracy of leaf-related traits in 291 maize inbred lines using the rrBLUP model. **(a)** The prediction accuracy estimated using different training population sizes. **(b)** The prediction accuracy estimated using different marker densities. LL1, length of the first leaf above the uppermost ear; LL2, length of the uppermost ear leaf; LL3, length of the first leaf below the uppermost ear; LW1, width of the first leaf above the uppermost ear; LW2, width of the uppermost ear leaf; LW3, width of the first leaf below the uppermost ear; LAr2, leaf area of the uppermost ear; LNAE, total number of leaves above the uppermost ear.

By using nine gradually increasing training population sizes, the prediction accuracy of the eight leaf-related traits exhibited a consistent trend: as the training population size increased, the prediction accuracy quickly increased and then gradually stabilized (**Figure 8a**; **Supplementary Table 6**). Once the training population size reached 70% of the total genotype, increasing training population size did not further improve prediction accuracy. When the training population size increased from 10% to 70%, the average prediction accuracy increased from 0.10 to 0.26 for LL1, 0.10 to 0.23 for LL2, 0.11 to 0.26 for LL3, 0.21 to 0.40 for LW1, 0.24 to 0.43 for LW2, 0.26 to 0.43 for LW3, 0.21 to 0.40 for LAr2, and 0.12 to 0.31 for LNAE.

By using eight gradually increasing marker densities, the prediction accuracy of the eight leaf-related traits exhibited a consistent trend: as the number of markers increased, the prediction accuracy quickly increased and then gradually stabilized at a marker density of 3000 (**Figure 8b**; **Supplementary Table 7**). By using 3000 SNPs for GS, the average prediction accuracy reached 0.21, 0.22, 0.26, 0.34, 0.36, 0.38, 0.34, and 0.24 for LL1, LL2, LL3, LW1, LW2, LW3, LAr2, and LNAE, respectively.

## 4 Discussion

### 4.1 Genetic architecture of leaf-related traits

Optimal leaf length, width, and area are essential for maximizing the capture of sunlight in maize, which in turn promotes seed development (Ballare et al., 2006). The phenotypic analysis of leaf-related traits across four environments revealed significant genotype variance and genotype-environment interaction variance. The medium to high heritability, ranged from 77.93% to 87.54%, indicating that leaf-related traits were mainly controlled by genetics. Our results were consistent with previous studies (Dai et al., 2023; Zeng et al., 2025). Dai et al. (2023) reported that the genotype, environment, and environment × genotype interaction all displayed significant effects on 25 leaf-related traits in 334 diverse maize inbreds, with heritability ranging from 83.61% to 91.68%. The results of Zeng et al. (2025) showed that there was a highly significant difference between the genotypic variance of leaf-related traits and the genotypic × environmental interaction variance (*P* < 0.001), with broad-sense heritabilities ranging from 0.73 to 0.80.

### 4.2 Significant SNPs for leaf-related traits

In this study, both FarmCPU and BLINK were used for association analysis of leaf-related traits. A total of 51 and 73 SNPs were detected by BLINK and FarmCPU at a stringent *p*-values threshold of 2.968 × 10^-6^, respectively. Eleven SNPs with pleiotropic effect located at bins 1.05, 1.07, 1.08, 3.05, 4.06, 6.01, 6.03, 6.07, 6.08, 7.02, and 9.02 were identified. Among the 11 pleiotropic SNPs, one SNP 1_85761290 was identified for the length of all the three examined leaves, indicating that the length of leaves located at different positions may be controlled by one or more common genetic loci. Eight SNPs were identified for two or three leaf width traits, indicating that the width of leaves at different positions of maize may share the same genetic characteristics. Similar results were reported previous (Guo et al., 2015; Ma et al., 2018; Nikolić et al., 2007). Guo et al. (2015) conducted QTL analysis for the width of four consecutive leaves located at different positions above the uppermost ear. Three, one, and six common QTLs were identified for all of the examined positions, three of the positions, and two of the positions, respectively. Ma et al. (2018) found three pairs of QTLs (*qLW-2–2* and *qLAr-2-1*, *qLW-8–1* and *qLL-8-2*, *qLL-3–3* and *qLAr-3-3*) in a QTL localization study of leaf-related traits in maize that control different traits and were located on or near the same chromosome. Nikolić et al. (2007) used a composite interval mapping method to analyze the QTL of yield traits and morphological traits, and found that the QTLs of TBN, LW3 and LW4 overlapped on chromosome 5.

In comparison with previous studies, seven of the 24 SNPs for leaf length have been supported. Two SNPs 7_117078358 and 7_129132515 located at bin 7.02 were mapped within the mapping interval of *sQTL19*, which was a stable QTL identified for leaf length in two different environments (Zhao et al., 2018). SNP 8_2077268 identified for LL1 by both methods was close to the SNPs SYN10052 and PUT-163a-13557719-256, which were identified for leaf length in 334 diverse maize inbred lines (Dai et al., 2023). The SNP 2_58557744 identified for LW3 by both methods was located 845,488 bp downstream of SNP PZE0259584444, which was identified in the maize nested association mapping population (Tian et al., 2011). SNP 3_97706527 associated with LL1 was located 883,926 bp downstream of SNP PZE0383801452 (Tian et al., 2011). SNP 8_2077268 identified for LL1 by both methods was located 986,469 bp upstream of SNP PZE0802740685 (Tian et al., 2011). SNP 10_151712093 associated with LL2 was 117,636 bp upstream of SNP PZE10149139260 (Tian et al., 2011).

Six of the 34 unique significant SNPs for leaf width were supported by comparisons with previous studies. The PVE of each SNP ranged from 0.01% to 17.20%. SNP 1_255445087 associated with LW3 and SNP 1_265225596 associated with LW2 were located at the mapping interval of *sQTL21*, which was a stable QTL identified for leaf width in four different populations across multiple environments (Zhao et al., 2018). SNPs 5_185308579 and 8_107035297 identified for LW1 were 500,021 bp and 776,350bp away from PZE-105128589 and SYN13530, respectively (Dai et al., 2023). SNP 5_185308579 associated with LW1 was located 500,021bp downstream of PZE-105128589 (Dai et al., 2023). SNP 8_107035297 associated with LW1 was located 776,350 bp upstream of SYN13530 (Dai et al., 2023). The pleiotropic SNP 6_178934629 identified for LW1, LW2, and LW3 was located 569,541 bp downstream of SNP PZE06167017453 (Tian et al., 2011).

Two of the 14 unique significant SNPs identified for LAr2 were consistent with previous studies. The PVE of each SNP ranged from 0.70% to 21.80%. The pleiotropic SNP 6_31979816 associated with LAr2 and LW3 was located 630,697 bp upstream of PZE-106009112, which was significantly associated with the eighth leaf area (Zhao et al., 2018). The pleiotropic SNP 6_31979816 identified for LAr2 and LW3 was located at the same bin 6.01 as *sQTL6*, which was a stable QTL for leaf angle detected in two environments (Zhao et al., 2018).

Four of the 19 unique significant SNPs identified for LNAE were supported by comparisons with previous studies. The SNP 4_230408762 consistently identified by both models for LNAE was located at the same bin 4.09 as *QLN4* (Hou et al., 2015). The SNP 10_139552005 for LNAE was located 618,250 bp upstream of PZE-110086343, which was associated with the first leaf area reported by Dai et al. (2023). SNPs 5_161516816 and 5_93495559 associated with LNAE were located in the same region as Chr5.S_174323150 in bin 5.04 (Liu et al., 2018). In conclusion, the present study detected abundant genetic loci affecting leaf growth and development. The stable and consistent SNPs identified in different models or traits should be given priority consideration in maize marker-assisted selection breeding.

### 4.3 Candidate genes retrieved through association analysis

Exploring candidate genes helps to better understand the genetic architecture of leaf-related traits. In this study, 19 candidate genes were annotated for the 11 pleiotropic SNPs. Of them, four candidate genes *Zm00001eb297330*, *Zm00001eb275550*, *Zm00001eb377180*, and *Zm00001eb296310* may play important roles in leaf growth and development. SNP 6_178934629 identified for LW1, LW2, and LW3, exhibits significant differences in leaf width between different haplotypes. *Zm00001eb297330*, the candidate gene of SNP 6_178934629, encodes a plant cysteine oxidase 2. Plant cysteine oxidases (*PCOs*) are oxygen-sensing enzymes that emit signals under hypoxic conditions following submergence, triggering adaptive responses in plants to survive under such stress (Taylor-Kearney and Flashman, 2022). They are considered potential oxygen sensors involved in regulating plant responses to hypoxia, and their gene expression varies according to changes in leaf size (White et al., 2017; Weits et al., 2014). In a study of Brassica napus, quantitative PCR (qPCR) results demonstrated that most members of *Bna/Bra/BoPCO5* exhibited relatively high gene expression in various parts of the plant, including flowers, leaves, and roots, suggesting that *PCOs* play an important role in the nutritional and reproductive development of plants (Bian et al., 2023).

*Zm00001eb275550*, the candidate gene for pleiotropic SNP 4_165604305, encodes a *STRUBBELIG* family receptor protein kinase. It is an atypical receptor kinase that regulates various developmental processes, including epidermal growth, leaf development, and flower morphogenesis (Chevalier et al., 2005; Kwak et al., 2005; Baute et al., 2015).

*Zm00001eb377180*, the candidate gene of pleiotropic SNP 9_21919877, encodes an *AP2*-like ethylene-responsive transcription factor. *AP2*/ethylene response factors play important roles in regulating plant growth, development, and stress responses, including leaf development and morphology (Zhang et al., 2022; Cheng et al., 2023).

*Zm00001eb296310*, the candidate gene of pleiotropic SNP 6_176813835, encodes a putative timeless C-term domain and Homeodomain protein. It was mapped to chromosome (GO:0005694) (P=0.044) and showed medium expression level in 30 days old leaves.

In addition, *Zm00001eb178140* with high expression levels in 30 days old leaves encodes a ribosomal protein L7-like protein, which is vital for regulating plant growth and development, particularly in leaf growth and the formation of leaf area. Ribosomal proteins are involved in cell proliferation and expansion, thereby affecting the overall growth potential of the plant (Luo et al., 2023). In conclusion, these candidate genes *Zm00001eb297330*, *Zm00001eb275550*, *Zm00001eb377180*, *Zm00001eb296310*, and *Zm00001eb178140* may play important roles in the configuration of maize leaf structures, which required further functional studies.

### 4.4 Genomic selection strategies for leaf-related traits

GS has emerged as a mainstream technology in the breeding of plants and animals, providing new insights and achieving significant results (Anilkumar et al., 2022). The GS prediction accuracy is influenced by many factors, including the training population size, marker density, trait heritability, genetic relationship between training population and prediction population, and prediction models (Liu et al., 2018). Previous studies have demonstrated that increasing the training population size and marker density can improve the prediction accuracy of GS. Dang et al. (2023) conducted GS on plant architecture traits in 190 sweet maize inbred lines and 287 waxy con inbred lines. The prediction accuracy increased rapidly, when the training population size increased from 10% to 30% or the number of marker density increased from 0 to 500. Then the prediction accuracy increased slightly. Fan et al. (2024) performed GS on flowering-related traits in a multi-parent DH population, a relatively high prediction accuracy was achieved with the training population size of 70% and marker density of 3000. In the study of Zeng et al. (2025), moderate prediction accuracy was obtained for leaf length, leaf width, and leaf area with the training population size of 60% and marker density of 3000.The prediction was significantly improved by using the top 300 significant SNPs by GWAS. Ren et al. (2021) conducted GS for common rust resistance in a GWAS panel and a bi-parental DH population. A relatively high prediction accuracy was achieved with the training population size of 50% in both population. The optimal marker density was 300 in the DH population and 5000 in the GWAS panel. Consistent with these results, as the training population size or marker density increased, the GS prediction accuracy of leaf-related traits quickly increased and then gradually stabilized. Once the training population size reached 70% of the total genotypes or the marker density reached 3000, the prediction reached a plateau. In combination with previous research findings, the optimal training population size for GS was 50%-70% of the total genotypes. However, the optimal marker density for GS depends on the LD decay distance of the population and population structure (Guo et al., 2020). The population with low LD decay distance requires more markers. In general, the optimal marker density is 300 to 500 in a bi-parental population and 3000 to 5000 in a GWAS panel (Ren et al., 2021; Zhang et al., 2017; Zeng et al., 2025; Fan et al., 2024). The prediction accuracy of leaf-related traits estimated through 5-fold cross-validation ranged from 0.22 to 0.44. More research is needed to improve the prediction accuracy of leaf-related traits.

## Data Availability

The datasets presented in this study can be found in online repositories. The names of the repository/repositories and accession number(s) can be found in the article/**Supplementary Material**.
