# Supplementary material for: Genome-wide association studies and genomic selection for leaf-related traits in maize
Source: Front Plant Sci. 2025 Dec 9;16:1669346. doi: 10.3389/fpls.2025.1669346 (PMC12731249; doi:10.3389/fpls.2025.1669346)
Supplement: Supplementary file 4 [file DataSheet4.docx]

Supplementary Material

# Supplementary Tables

| **Supplementary Table 1.** Significant SNPs associated with leaf-related traits identified in 291 maize inbred lines based on BLINK and FarmCPU models. | | | | | | | |
| --- | --- | --- | --- | --- | --- | --- | --- |
| Trait^1^ | Model | SNP^2^ | P-value | Allele | Maf | Bin^3^ | PVE^4^（%） |
| LL1 | BLINK | 3_97706527 | 1.52 × 10^-6^ | G/A | 0.09 | 3.04 | 6.03 |
|  |  | 7_117078358 | 2.05 × 10^-9^ | A/G | 0.05 | 7.02 | 12.60 |
|  |  | 7_129132515 | 7.37 × 10^-8^ | C/T | 0.05 | 7.02 | 13.70 |
|  |  | 8_2077268 | 1.61 × 10^-11^ | G/A | 0.07 | 8.00 | 16.10 |
|  |  | 9_106240229 | 1.84 × 10^-6^ | G/T | 0.05 | 9.04 | 6.25 |
|  |  | 9_122936513 | 3.41 × 10^-8^ | C/T | 0.09 | 9.04 | 7.11 |
|  |  | 10_25646435 | 4.13 × 10^-8^ | A/C | 0.05 | 10.03 | 14.8 |
|  |  | 10_98449512 | 2.95 × 10^-6^ | G/A | 0.43 | 10.04 | 4.82 |
|  | FarmCPU | 1_85761290 | 5.66 × 10^-7^ | C/T | 0.05 | 1.05 | 11.80 |
|  |  | 8_2077268 | 8.43 × 10^-8^ | G/A | 0.07 | 8.00 | 14.90 |
|  |  | 8_156179569 | 2.38 × 10^-6^ | A/G | 0.08 | 8.06 | 13.20 |
|  |  | 9_122936513 | 4.17 × 10^-7^ | C/T | 0.09 | 9.04 | 19.20 |
| LL2 | BLINK | 1_159903081 | 2.18 × 10^-10^ | C/A | 0.07 | 1.05 | 17.90 |
|  |  | 3_17054552 | 5.71 × 10^-10^ | C/T | 0.05 | 3.04 | 20.70 |
|  |  | 3_95871968 | 6.89 × 10^-12^ | C/T | 0.08 | 3.04 | 14.10 |
|  |  | 5_187913975 | 2.17 × 10^-6^ | C/A | 0.11 | 5.05 | 8.00 |
|  |  | 10_151712093 | 4.07 × 10^-9^ | G/A | 0.11 | 10.07 | 11.20 |
|  | FarmCPU | 1_85761290 | 1.01 × 10^-6^ | C/T | 0.05 | 1.05 | 11.70 |
|  |  | 1_159903081 | 4.58 × 10^-7^ | C/A | 0.07 | 1.05 | 14.80 |
|  |  | 3_95871968 | 8.62 × 10^-8^ | C/T | 0.08 | 3.04 | 16.90 |
|  |  | 4_53255003 | 2.94 × 10^-6^ | A/G | 0.06 | 4.05 | 13.50 |
| LL3 | BLINK | 2_58557744 | 1.85 × 10^-11^ | G/A | 0.09 | 2.04 | 12.70 |
|  |  | 3_187354520 | 8.23 × 10^-9^ | T/C | 0.07 | 3.06 | 14.30 |
|  |  | 4_72809458 | 6.35 × 10^-8^ | A/G | 0.07 | 4.05 | 11.60 |
|  |  | 6_51873235 | 4.92 × 10^-8^ | A/C | 0.06 | 6.01 | 15.30 |
|  |  | 8_144381051 | 1.57 × 10^-9^ | C/T | 0.09 | 8.05 | 9.37 |
|  |  | 9_89375725 | 1.33 × 10^-6^ | A/T | 0.05 | 9.03 | 14.30 |
|  | FarmCPU | 1_85761290 | 1.57 × 10^-7^ | C/T | 0.05 | 1.05 | 8.23 |
|  |  | 2_58557744 | 9.37 × 10^-8^ | G/A | 0.09 | 2.04 | 8.31 |
|  |  | 3_187354520 | 3.97 × 10^-7^ | T/C | 0.07 | 3.06 | 14.60 |
|  |  | 6_31513842 | 9.42 × 10^-7^ | T/C | 0.05 | 6.01 | 15.80 |
|  |  | 6_148137933 | 1.86 × 10^-6^ | C/T | 0.06 | 6.05 | 7.01 |
|  |  | 8_144381051 | 5.24 × 10^-7^ | C/T | 0.09 | 8.05 | 6.99 |
| LW1 | BLINK | 1_163279435 | 2.21 × 10^-6^ | G/A | 0.06 | 1.05 | 12.80 |
|  |  | 1_204491759 | 6.14 × 10^-9^ | A/T | 0.41 | 1.07 | 4.10 |
|  |  | 6_92939142 | 1.34 × 10^-6^ | A/C | 0.18 | 6.01 | 4.19 |
|  |  | 6_107526600 | 4.11 × 10^-10^ | T/C | 0.06 | 6.03 | 16.90 |
|  |  | 6_178934629 | 8.58 × 10^-10^ | C/T | 0.48 | 6.08 | 8.46 |
|  |  | 8_107035297 | 6.17 × 10^-9^ | T/A | 0.47 | 8.03 | 15.90 |
|  |  | 9_21919877 | 2.12 × 10^-6^ | T/C | 0.12 | 9.02 | 7.00 |
|  | FarmCPU | 1_204491759 | 9.25 × 10^-7^ | T/A | 0.41 | 1.07 | 2.66 |
|  |  | 1_228170725 | 2.63 × 10^-6^ | G/A | 0.06 | 1.07 | 2.17 |
|  |  | 3_220511695 | 6.75 × 10^-7^ | A/G | 0.16 | 3.08 | 5.77 |
|  |  | 4_154773982 | 1.48 × 10^-6^ | C/T | 0.12 | 4.05 | 8.50 |
|  |  | 4_165604305 | 1.20 × 10^-6^ | C/T | 0.09 | 4.06 | 0.01 |
|  |  | 5_185308579 | 2.07 × 10^-6^ | G/A | 0.08 | 5.05 | 0.01 |
|  |  | 6_107526600 | 2.91 × 10^-7^ | C/T | 0.06 | 6.03 | 1.17 |
|  |  | 6_176813835 | 1.47 × 10^-6^ | G/T | 0.05 | 6.07 | 8.37 |
|  |  | 6_178934629 | 1.09 × 10^-7^ | T/C | 0.48 | 6.08 | 5.36 |
|  |  | 6_180292384 | 1.75 × 10^-6^ | G/A | 0.09 | 6.08 | 1.07 |
|  |  | 7_130100281 | 7.68 × 10^-7^ | G/T | 0.05 | 7.02 | 0.01 |
|  |  | 7_177549605 | 1.30 × 10^-6^ | C/T | 0.08 | 7.05 | 1.13 |
|  |  | 9_21919877 | 4.67 × 10^-7^ | T/C | 0.12 | 9.02 | 5.73 |
| LW2 | BLINK | 1_184376050 | 2.80 × 10^-6^ | T/C | 0.50 | 1.06 | 13.20 |
|  |  | 2_55812778 | 5.46 × 10^-8^ | G/A | 0.10 | 2.04 | 9.35 |
|  |  | 3_150800464 | 6.22 × 10^-7^ | C/T | 0.07 | 3.05 | 7.90 |
|  |  | 4_142450042 | 3.51 × 10^-9^ | T/C | 0.05 | 4.05 | 17.00 |
|  |  | 6_176813835 | 8.45 × 10^-9^ | T/G | 0.05 | 6.07 | 12.10 |
|  |  | 6_178934629 | 7.67 × 10^-14^ | C/T | 0.48 | 6.08 | 4.94 |
|  |  | 9_21919877 | 3.63 × 10^-8^ | C/T | 0.12 | 9.02 | 7.71 |
|  |  | 10_151817446 | 1.04 × 10^-6^ | A/G | 0.07 | 10.07 | 8.47 |
|  | FarmCPU | 1_265225596 | 4.56 × 10^-8^ | C/G | 0.50 | 1.09 | 1.31 |
|  |  | 2_28091358 | 2.70 × 10^-8^ | C/T | 0.50 | 2.03 | 6.32 |
|  |  | 2_57643486 | 1.75 × 10^-8^ | T/G | 0.25 | 2.04 | 2.35 |
|  |  | 2_148710460 | 1.12 × 10^-7^ | G/A | 0.05 | 2.05 | 11.50 |
|  |  | 2_203013088 | 7.68 × 10^-8^ | G/A | 0.13 | 2.07 | 5.15 |
|  |  | 3_150800464 | 8.27 × 10^-7^ | T/C | 0.07 | 3.05 | 3.79 |
|  |  | 3_191038224 | 2.45 × 10^-8^ | A/T | 0.12 | 3.06 | 3.22 |
|  |  | 5_155798109 | 1.89 × 10^-8^ | T/A | 0.05 | 5.04 | 8.34 |
|  |  | 5_158965347 | 2.71 × 10^-6^ | A/T | 0.18 | 5.04 | 3.21 |
|  |  | 6_178934629 | 1.51 × 10^-14^ | T/C | 0.48 | 6.08 | 3.81 |
|  |  | 7_68627719 | 8.34 × 10^-9^ | C/T | 0.07 | 7.02 | 8.69 |
|  |  | 7_130100281 | 2.84 × 10^-8^ | G/T | 0.04 | 7.02 | 15.30 |
|  |  | 9_21919877 | 3.05 × 10^-7^ | T/C | 0.12 | 9.02 | 3.34 |
|  |  | 9_44938600 | 1.07 × 10^-6^ | A/G | 0.12 | 9.03 | 3.29 |
| LW3 | BLINK | 1_204491759 | 3.90 × 10^-9^ | A/T | 0.41 | 1.07 | 5.05 |
|  |  | 5_213046877 | 9.06 × 10^-7^ | T/C | 0.21 | 5.07 | 4.56 |
|  |  | 6_107526600 | 9.60 × 10^-10^ | T/C | 0.06 | 6.03 | 17.20 |
|  |  | 6_178934629 | 3.54 × 10^-10^ | C/T | 0.48 | 6.08 | 12.30 |
|  | FarmCPU | 1_204491759 | 6.29 × 10^-7^ | T/A | 0.41 | 1.07 | 4.06 |
|  |  | 1_255445087 | 5.18 × 10^-7^ | T/G | 0.26 | 1.08 | 3.24 |
|  |  | 3_150800464 | 1.29 × 10^-6^ | T/C | 0.07 | 3.05 | 5.93 |
|  |  | 4_165604305 | 2.30 × 10^-6^ | C/T | 0.09 | 4.06 | 2.56 |
|  |  | 6_31979816 | 2.04 × 10^-6^ | G/A | 0.05 | 6.01 | 7.05 |
|  |  | 6_107526600 | 2.63 × 10^-7^ | C/T | 0.06 | 6.03 | 6.74 |
|  |  | 6_176813835 | 2.72 × 10^-6^ | G/T | 0.05 | 6.07 | 9.04 |
|  |  | 6_178934629 | 2.42 × 10^-7^ | T/C | 0.48 | 6.08 | 7.24 |
| LAr2 | BLINK | 2_31225540 | 1.37 × 10^-8^ | T/C | 0.10 | 2.04 | 12.00 |
|  |  | 2_158371143 | 6.60 × 10^-9^ | T/A | 0.48 | 2.06 | 4.83 |
|  |  | 3_155422447 | 2.09 × 10^-6^ | G/A | 0.05 | 3.05 | 15.00 |
|  |  | 4_6296569 | 1.29 × 10^-6^ | C/A | 0.05 | 4.02 | 6.72 |
|  |  | 6_31979816 | 2.48 × 10^-8^ | A/G | 0.05 | 6.01 | 21.80 |
|  |  | 8_63285721 | 1.79 × 10^-6^ | C/T | 0.07 | 8.03 | 19.10 |
|  |  | 9_115836774 | 1.19 × 10^-6^ | T/G | 0.24 | 9.04 | 2.86 |
|  | FarmCPU | 1_82290974 | 1.83 × 10^-6^ | A/G | 0.18 | 1.04 | 4.89 |
|  |  | 1_255445087 | 2.53 × 10^-6^ | T/G | 0.26 | 1.08 | 1.38 |
|  |  | 2_31225540 | 3.00 × 10^-7^ | C/T | 0.10 | 2.04 | 13.90 |
|  |  | 2_210216699 | 2.09 × 10^-6^ | C/T | 0.50 | 2.07 | 9.45 |
|  |  | 4_172381592 | 1.46 × 10^-6^ | A/G | 0.09 | 4.06 | 1.97 |
|  |  | 4_185632929 | 5.66 × 10^-7^ | C/T | 0.11 | 4.08 | 0.70 |
|  |  | 6_31979816 | 3.91 × 10^-7^ | G/A | 0.05 | 6.01 | 15.10 |
|  |  | 6_107526600 | 2.10 × 10^-6^ | C/T | 0.06 | 6.03 | 10.70 |
|  |  | 10_139944323 | 1.51 × 10^-6^ | A/T | 0.14 | 10.05 | 4.81 |
| LNAE | BLINK | 2_28230945 | 2.63 × 10^-8^ | T/G | 0.09 | 2.03 | 8.65 |
|  |  | 4_157618103 | 1.78 × 10^-9^ | T/A | 0.07 | 4.06 | 25.01 |
|  |  | 4_230408762 | 9.17 × 10^-8^ | T/C | 0.07 | 4.09 | 12.40 |
|  |  | 5_93495559 | 2.97 × 10^-12^ | A/G | 0.09 | 5.04 | 13.40 |
|  |  | 7_13474867 | 1.97 × 10^-6^ | C/T | 0.10 | 7.01 | 5.12 |
|  |  | 10_139552005 | 1.51 × 10^-6^ | G/A | 0.05 | 10.05 | 13.10 |
|  | FarmCPU | 1_121290934 | 2.34 × 10^-6^ | T/C | 0.10 | 1.05 | 1.21 |
|  |  | 2_203764800 | 1.32 × 10^-7^ | C/T | 0.08 | 2.07 | 7.61 |
|  |  | 3_66979466 | 2.08 × 10^-8^ | C/T | 0.06 | 3.04 | 8.18 |
|  |  | 3_231932999 | 4.53 × 10^-7^ | A/C | 0.15 | 3.09 | 2.74 |
|  |  | 4_54399833 | 8.47 × 10^-7^ | A/C | 0.50 | 4.05 | 2.54 |
|  |  | 4_68805902 | 1.25 × 10^-8^ | C/A | 0.11 | 4.05 | 8.96 |
|  |  | 4_230408762 | 4.68 × 10^-13^ | C/T | 0.07 | 4.09 | 14.60 |
|  |  | 5_22515216 | 9.49 × 10^-7^ | C/A | 0.06 | 5.03 | 6.49 |
|  |  | 5_93495559 | 1.89 × 10^-9^ | G/A | 0.09 | 5.04 | 6.62 |
|  |  | 5_161516816 | 4.79 × 10^-8^ | T/C | 0.13 | 5.04 | 3.11 |
|  |  | 6_7600666 | 3.18 × 10^-7^ | A/G | 0.07 | 6.00 | 6.07 |
|  |  | 7_134339386 | 2.46 × 10^-11^ | C/G | 0.12 | 7.03 | 4.17 |
|  |  | 8_5783359 | 8.27 × 10^-7^ | A/T | 0.06 | 8.01 | 3.10 |
|  |  | 8_107435853 | 9.58 × 10^-7^ | G/T | 0.05 | 8.03 | 5.97 |
|  |  | 10_112779464 | 1.67 × 10^-6^ | A/G | 0.09 | 10.04 | 4.32 |

^1^ LL1: length of the first leaf above the uppermost ear; LL2: length of the uppermost ear leaf; LL3: length of the first leaf below the uppermost ear; LW1: width of the first leaf above the uppermost ear; LW2: width of the uppermost ear leaf; LW3: width of the first leaf below the uppermost ear; LAr2: leaf area of the uppermost ear; LNAE: total number of leaves above the uppermost ear. ^2^ SNP names, chromosome position, 3_97706527 refers that the SNP is located on chromosome 3 with the physical position of 97706527 bp. ^3^ Chromosomal region. ^4^ PVE: phenotypic variance explained by each SNP.

| **Supplementary Table 2.** Functional annotation of 122 candidate genes identified for leaf-related traits in 291 maize inbred lines. | | | | |
| --- | --- | --- | --- | --- |
| SNP^a^ | Trait^b^ | Gene ID^c^ | Bin^d^ | Gene annotation^e^ |
| 7_117078358 | LL1 | Zm00001eb312600 | 7.02 | MYB-transcription factor 162 |
| 8_2077268 | LL1 | Zm00001eb332610 | 8.00 | Major facilitator superfamily (MFS) profile domain-containing protein |
| 9_122936513 | LL1 | Zm00001eb391790 | 9.04 | C2 DOCK-type domain-containing protein |
| 10_25646435 | LL1 | Zm00001eb410510 | 10.03 | E3 ubiquitin-protein ligase MIEL1 |
| 1_159903081 | LL2 | Zm00001eb029450 | 1.05 | vacuolar amino acid transporter 1 |
| 3_17054552 | LL2 | Zm00001eb124190 | 3.04 | Hydroxycinnamoyltransferase7 |
|  |  | Zm00001eb124200 | 3.04 | HMG aldolase(Oxaloacetate decarboxylase) |
| 3_95871968 | LL2 | Zm00001eb133100 | 3.04 | Bidirectional sugar transporter SWEET |
| 10_151712093 | LL2 | Zm00001eb434460 | 10.07 | 30S ribosomal protein S17, chloroplastic |
| 4_72809458 | LL3 | Zm00001eb178140 | 4.05 | 60S ribosomal protein L7-like |
|  |  | Zm00001eb178150 | 4.05 | Uncharacterized protein |
| 6_51873235 | LL3 | Zm00001eb267500 | 6.01 | Leucine-rich repeat (LRR) family protein |
| 8_144381051 | LL3 | Zm00001eb357550 | 8.05 | Uncharacterized protein |
| 1_204491759 | LW1、LW3 | Zm00001eb038260 | 1.07 | Uncharacterized protein |
|  |  | Zm00001eb038270 | 1.07 | Patatin-like protein 2-like |
| 6_107526600 | LW1、LW3 | Zm00001eb275550 | 6.03 | STRUBBELIG family receptor protein kinase |
|  |  | Zm00001eb275560 | 6.03 | Nop domain-containing protein |
|  |  | Zm00001eb275570 | 6.03 | Nudix hydrolase 2-like |
| 6_178934629 | LW1、LW2、LW3 | Zm00001eb297330 | 6.08 | Plant cysteine oxidase 2 |
| 8_107035297 | LW1 | Zm00001eb349450 | 8.03 | MYB DNA-binding domain superfamily protein |
|  |  | Zm00001eb349460 | 8.03 | MYB DNA-binding domain superfamily protein |
| 2_55812778 | LW2 | Zm00001eb083180 | 2.04 | Uncharacterized protein |
| 4_142450042 | LW2 | Zm00001eb184560 | 4.05 | Sister chromatid cohesion protein PDS5 B-B |
| 6_176813835 | LW2 | Zm00001eb296310 | 6.07 | putative timeless C-term domain and Homeodomain protein |
|  |  | Zm00001eb296320 | 6.07 | DUF3143 family protein |
|  |  | Zm00001eb296330 | 6.07 | Uncharacterized protein |
|  |  | Zm00001eb296340 | 6.07 | G-box binding factor 1 |
|  |  | Zm00001eb296350 | 6.07 | glucose-6-phosphate 1-epimerase (EC 5.1.3.15) |
|  |  | Zm00001eb296360 | 6.07 | Uncharacterized protein |
|  |  | Zm00001eb296370 | 6.07 | Uncharacterized protein |
| 9_21919877 | LW1、LW2 | Zm00001eb377180 | 9.02 | AP2-EREBP-transcription factor 33 |
| 2_31225540 | LAr2 | Zm00001eb077760 | 2.04 | Coiled-coil domain-containing protein 115 |
|  |  | Zm00001eb077770 | 2.04 | Receptor-like serine/threonine-protein kinase ALE2 |
|  |  | Zm00001eb077780 | 2.04 | DEAD-box ATP-dependent RNA helicase 30 |
| 2_158371143 | LAr2 | Zm00001eb094370 | 2.06 | probable serine/threonine-protein kinase DDB_G0276461 |
| 2_28230945 | LNAE | Zm00001eb076710 | 2.03 | Cox19-like CHCH family protein |
|  |  | Zm00001eb076720 | 2.03 | Mitochondrial substrate carrier family protein |
|  |  | Zm00001eb076730 | 2.03 | Endosomal targeting BRO1-like domain-containing protein |
|  |  | Zm00001eb076740 | 2.03 | FIP1 |
|  |  | Zm00001eb076750 | 2.03 | putative GEM-like protein 8 |
| 4_230408762 | LNAE | Zm00001eb203860 | 4.09 | F-box/kelch-repeat protein |
| 5_93495559 | LNAE | Zm00001eb233990 | 5.04 | Uncharacterized protein |
| 7_130100281 | LW1、LW2 | Zm00001eb314670 | 7.02 | Beta-1,3-galactosyltransferase 7 |
| 1_265225596 | LW2 | Zm00001eb052570 | 1.09 | Auxin responsive protein |
|  |  | Zm00001eb052580 | 1.09 | Auxin responsive protein |
| 2_28091358 | LW2 | Zm00001eb076670 | 2.03 | Protein CHROMATIN REMODELING 24 |
|  |  | Zm00001eb076680 | 2.03 | Protein STAY-GREEN LIKE chloroplastic |
|  |  | Zm00001eb076690 | 2.03 | probable aquaporin TIP3-2 |
|  |  | Zm00001eb076700 | 2.03 | Uncharacterized protein |
| 2_57643486 | LW2 | Zm00001eb083590 | 2.04 | transcription factor E2FA |
|  |  | Zm00001eb083600 | 2.04 | Transmembrane protein C20orf108 |
|  |  | Zm00001eb083610 | 2.04 | Non-specific lipid-transfer protein |
| 2_203013088 | LW2 | Zm00001eb103880 | 2.07 | DUF1279 domain-containing protein |
|  |  | Zm00001eb103890 | 2.07 | H/ACA ribonucleoprotein complex subunit 1 |
|  |  | Zm00001eb103900 | 2.07 | Thioredoxin superfamily protein |
|  |  | Zm00001eb103910 | 2.07 | leucine aminopeptidase |
|  |  | Zm00001eb103920 | 2.07 | Uncharacterized protein |
|  |  | Zm00001eb103930 | 2.07 | Leukotriene A-4 hydrolase-like protein |
| 3_191038224 | LW2 | Zm00001eb149740 | 3.06 | GTP binding protein |
|  |  | Zm00001eb149750 | 3.06 | TLC domain-containing protein 4-B |
|  |  | Zm00001eb149760 | 3.06 | Calcium uniporter protein 6, mitochondrial-like |
| 5_155798109 | LW2 | Zm00001eb239730 | 5.04 | Uncharacterized protein |
| 1_255445087 | LW3 | Zm00001eb050120 | 1.08 | Os03g0604500-like protein |
|  |  | Zm00001eb050130 | 1.08 | Serine-threonine protein kinase plant-type |
|  |  | Zm00001eb050140 | 1.08 | subtilisin-like protease |
| 4_185632929 | LAr2 | Zm00001eb194170 | 4.08 | TPR repeat-containing thioredoxin TTL4 |
| 2_203764800 | LNAE | Zm00001eb104210 | 2.07 | BZIP transcription factor |
|  |  | Zm00001eb104220 | 2.07 | 60 ribosomal protein L14 |
|  |  | Zm00001eb104230 | 2.07 | Nuclear pore complex protein NUP96 |
|  |  | Zm00001eb104240 | 2.07 | Endoplasmic reticulum vesicle transporter C-terminal domain-containing protein |
| 3_66979466 | LNAE | Zm00001eb131550 | 3.04 | thiamin pyrophosphokinase 1 |
| 5_22515216 | LNAE | Zm00001eb220070 | 5.03 | Uncharacterized protein |
|  |  | Zm00001eb220090 | 5.03 | Protein HLB1-like |
|  |  | Zm00001eb220100 | 5.03 | Protein kinase domain-containing protein |
|  |  | Zm00001eb220110 | 5.03 | Cytochrome b5 |
| 5_161516816 | LNAE | Zm00001eb240560 | 5.04 | UDP-glycosyltransferases domain-containing protein |
|  |  | Zm00001eb240570 | 5.04 | Uncharacterized protein |
|  |  | Zm00001eb240580 | 5.04 | UDP-glycosyltransferase 73D1 |
|  |  | Zm00001eb240590 | 5.04 | Glycosyltransferase (EC 2.4.1.-) |
| 7_134339386 | LNAE | Zm00001eb315650 | 7.03 | Blue copper protein |
|  |  | Zm00001eb315660 | 7.03 | transcriptional regulatory protein algP |
| 4_165604305 | LW1、LW3 | Zm00001eb188530 | 4.06 | Carbamoyl-phosphate synthase |
| 9_106240229 | LL1 | Zm00001eb388240 | 9.04 | Polyphenol oxidase, chloroplastic |
| 10_98449512 | LL1 | Zm00001eb419130 | 10.04 | protein BREAST CANCER SUSCEPTIBILITY 2 homolog A |
| 5_187913975 | LL2 | Zm00001eb247060 | 5.05 | UDP-N-acetylglucosamine transporter UGNT1 |
| 6_92939142 | LW1 | Zm00001eb272160 | 6.01 | nucleotide binding protein |
|  |  | Zm00001eb272170 | 6.01 | zinc-finger DNL superfamily protein |
|  |  | Zm00001eb272180 | 6.01 | DUF616 superfamily protein |
|  |  | Zm00001eb272190 | 6.01 | Uncharacterized protein |
| 1_184376050 | LW2 | Zm00001eb033030 | 1.06 | DIMBOA UDP-glucosyltransferase BX9 |
| 10_151817446 | LW2 | Zm00001eb434490 | 10.07 | BTB/POZ domain-containing protein TNFAIP1 |
|  |  | Zm00001eb434500 | 10.07 | RING/FYVE/PHD zinc finger superfamily protein |
| 3_155422447 | LAr2 | Zm00001eb141710 | 3.05 | zinc finger (C3HC4-type RING finger) family protein |
|  |  | Zm00001eb141720 | 3.05 | uncharacterized protein LOC100277262 |
| 4_6296569 | LAr2 | Zm00001eb166700 | 4.02 | Monosaccharide-sensing protein 2 |
|  |  | Zm00001eb166710 | 4.02 | Oligouridylate-binding protein 1B |
|  |  | Zm00001eb166740 | 4.02 | Invertebrate defensins family profile domain-containing |
|  |  | Zm00001eb166750 | 4.02 | Remorin family protein |
| 9_115836774 | LAr2 | Zm00001eb390000 | 9.04 | Uncharacterized protein |
| 7_13474867 | LNAE | Zm00001eb302140 | 7.01 | Thioredoxin H-type |
| 10_139552005 | LNAE | Zm00001eb428710 | 10.05 | Phosphoenolpyruvate carboxylase |
|  |  | Zm00001eb428720 | 10.05 | Uncharacterized protein |
|  |  | Zm00001eb428740 | 10.05 | outer cell layer 2 |
|  |  | Zm00001eb428750 | 10.05 | Replication factor C subunit 4 |
| 8_156179569 | LL1 | Zm00001eb360680 | 8.06 | Uncharacterized protein |
|  |  | Zm00001eb360690 | 8.06 | Phytochrome-associated serine/threonine-protein phosphatase-like |
|  |  | Zm00001eb360700 | 8.06 | uncharacterized protein LOC100275237 |
| 6_148137933 | LL3 | Zm00001eb285360 | 6.05 | dehydrin DHN1 |
| 4_154773982 | LW1 | Zm00001eb186150 | 4.05 | B box-type domain-containing protein |
| 5_185308579 | LW1 | Zm00001eb246380 | 5.05 | Helitron helicase-like domain-containing protein |
|  |  | Zm00001eb246390 | 5.05 | pentatricopeptide repeat-containing protein At3g04130, mitochondrial |
|  |  | Zm00001eb246400 | 5.05 | Ankyrin repeat family protein |
| 7_177549605 | LW1 | Zm00001eb328690 | 7.05 | ribonuclease P |
|  |  | Zm00001eb328700 | 7.05 | dirigent protein 6-like |
| 1_82290974 | LAr2 | Zm00001eb021610 | 1.04 | glutathione S-transferase GST 32 |
|  |  | Zm00001eb021620 | 1.04 | glutathione transferase40 |
| 2_210216699 | LAr2 | Zm00001eb106120 | 2.07 | phospholipase A I |
|  |  | Zm00001eb106130 | 2.07 | glutamate receptor 3.4 |
|  |  | Zm00001eb106140 | 2.07 | Septum-promoting GTP-binding protein 1 |
| 10_139944323 | LAr2 | Zm00001eb428860 | 10.05 | NAC domain-containing protein |
|  |  | Zm00001eb428870 | 10.05 | aluminum-activated malate transporter 10 |
|  |  | Zm00001eb428880 | 10.06 | Spc97 / Spc98 family of spindle pole body (SBP) component |

^a^ SNP name, chromosome_position, 7_117078358 refers that the SNP is located on chromosome 7 with the physical position of 117078358 bp. ^b^ LL1: length of the first leaf above the uppermost ear; LL2: length of the uppermost ear leaf; LL3: length of the first leaf below the uppermost ear; LW1: width of the first leaf above the uppermost ear; LW2: width of the uppermost ear leaf; LW3: width of the first leaf below the uppermost ear; LAr2: leaf area of the uppermost ear; LNAE: total number of leaves above the uppermost ear. ^c^ Putative candidate genes based on B73_RefGen_v5_genomic reference genome. ^d^ Chromosomal region. ^e^ Functional annotation information for gene in NCBI.

| **Supplementary Table 6.** GS prediction accuracy of leaf-related traits under different training population sizes using the rrBLUP model. | | | | | | | | |
| --- | --- | --- | --- | --- | --- | --- | --- | --- |
| Training population size | LL1^a^ | LL2^b^ | LL3^c^ | LW1^d^ | LW2^e^ | LW3^f^ | LAr2^g^ | LNAE^h^ |
| 10% | 0.10 | 0.10 | 0.11 | 0.21 | 0.24 | 0.26 | 0.21 | 0.12 |
| 20% | 0.14 | 0.13 | 0.13 | 0.28 | 0.31 | 0.33 | 0.27 | 0.18 |
| 30% | 0.17 | 0.13 | 0.16 | 0.30 | 0.35 | 0.36 | 0.31 | 0.20 |
| 40% | 0.19 | 0.15 | 0.18 | 0.33 | 0.36 | 0.38 | 0.34 | 0.23 |
| 50% | 0.20 | 0.17 | 0.20 | 0.35 | 0.38 | 0.40 | 0.36 | 0.25 |
| 60% | 0.21 | 0.20 | 0.22 | 0.37 | 0.40 | 0.42 | 0.38 | 0.26 |
| 70% | 0.26 | 0.23 | 0.26 | 0.40 | 0.43 | 0.43 | 0.40 | 0.31 |
| 80% | 0.25 | 0.22 | 0.25 | 0.41 | 0.44 | 0.44 | 0.41 | 0.31 |
| 90% | 0.25 | 0.24 | 0.25 | 0.40 | 0.43 | 0.43 | 0.40 | 0.31 |

^a^ LL1: length of the first leaf above the uppermost ear; ^b^ LL2: length of the uppermost ear leaf; ^c^ LL3: length of the first leaf below the uppermost ear; ^d^ LW1: width of the first leaf above the uppermost ear; ^e^ LW2: width of the uppermost ear leaf; ^f^ LW3: width of the first leaf below the uppermost ear;

^g^ LAr2: leaf area of the uppermost ear; ^h^ LNAE: total number of leaves above the uppermost ear.

| **Supplementary Table 7.** GS prediction accuracy of leaf-related traits under different marker densities using the rrBLUP model and 5-fold cross-validation method. | | | | | | | | |
| --- | --- | --- | --- | --- | --- | --- | --- | --- |
| Number of SNPs | LL1^a^ | LL2^b^ | LL3^c^ | LW1^d^ | LW2^e^ | LW3^f^ | LAr2^g^ | LNAE^h^ |
| 100 | 0.1 | 0.09 | 0.10 | 0.13 | 0.15 | 0.16 | 0.13 | 0.12 |
| 300 | 0.15 | 0.14 | 0.14 | 0.19 | 0.23 | 0.23 | 0.20 | 0.17 |
| 500 | 0.16 | 0.16 | 0.19 | 0.22 | 0.26 | 0.25 | 0.22 | 0.18 |
| 1000 | 0.19 | 0.18 | 0.24 | 0.26 | 0.30 | 0.30 | 0.27 | 0.21 |
| 3000 | 0.21 | 0.22 | 0.26 | 0.34 | 0.36 | 0.38 | 0.34 | 0.24 |
| 5000 | 0.22 | 0.23 | 0.28 | 0.35 | 0.38 | 0.40 | 0.36 | 0.25 |
| 10000 | 0.22 | 0.24 | 0.29 | 0.36 | 0.39 | 0.40 | 0.36 | 0.26 |
| 30000 | 0.23 | 0.24 | 0.30 | 0.38 | 0.39 | 0.41 | 0.38 | 0.26 |

^a^ LL1: length of the first leaf above the uppermost ear; ^b^ LL2: length of the uppermost ear leaf; ^c^ LL3: length of the first leaf below the uppermost ear; ^d^ LW1: width of the first leaf above the uppermost ear; ^e^ LW2: width of the uppermost ear leaf; ^f^ LW3: width of the first leaf below the uppermost ear;

^g^ LAr2: leaf area of the uppermost ear; ^h^ LNAE: total number of leaves above the uppermost ear.

# Supplementary Figures


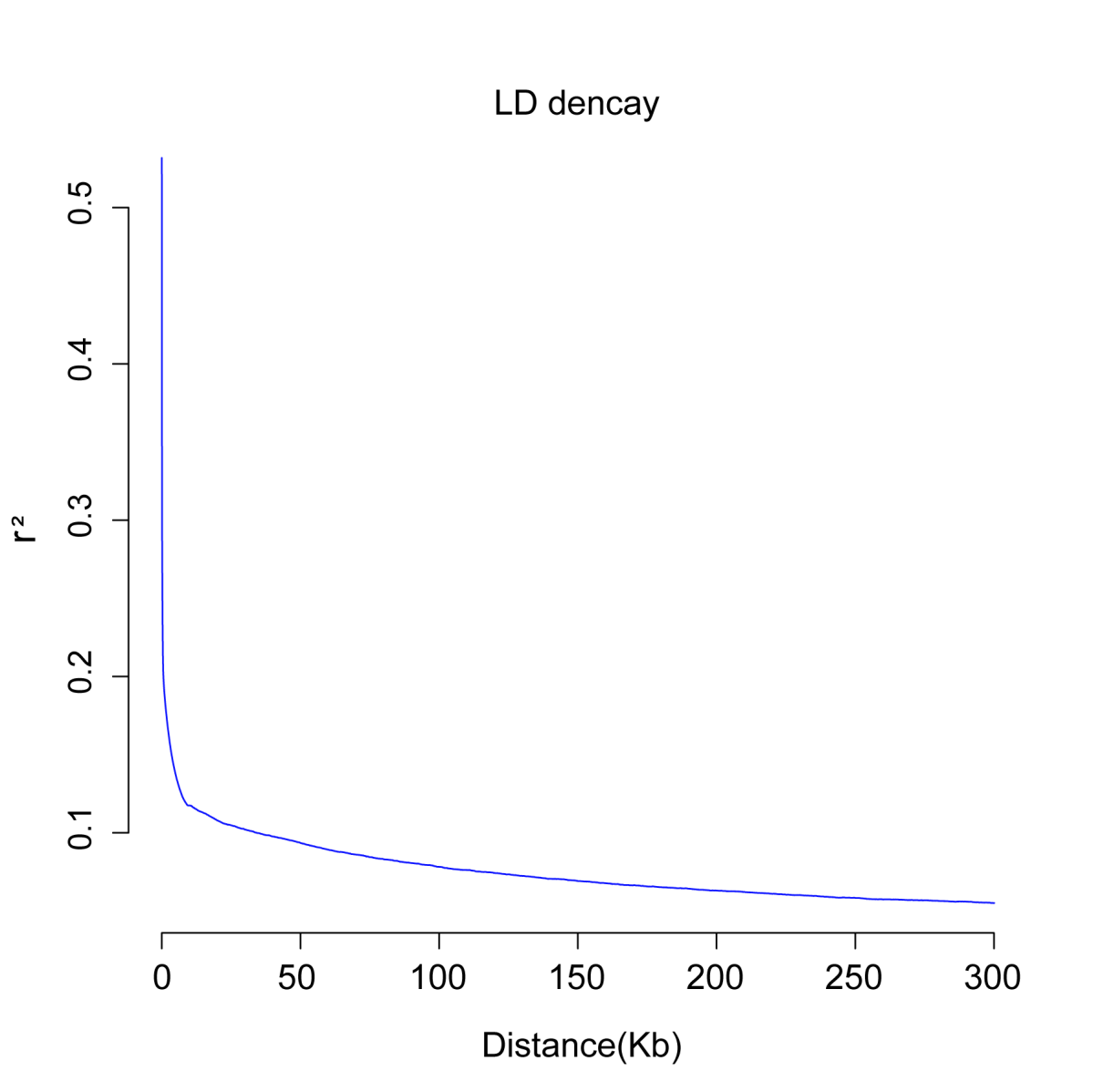


**Supplementary Figure 1** Linkage disequilibrium decay plot of the GWAS panel. The x-axis represents the physical distance between markers (kb), while the y-axis shows the average LD coefficient (r^2^).


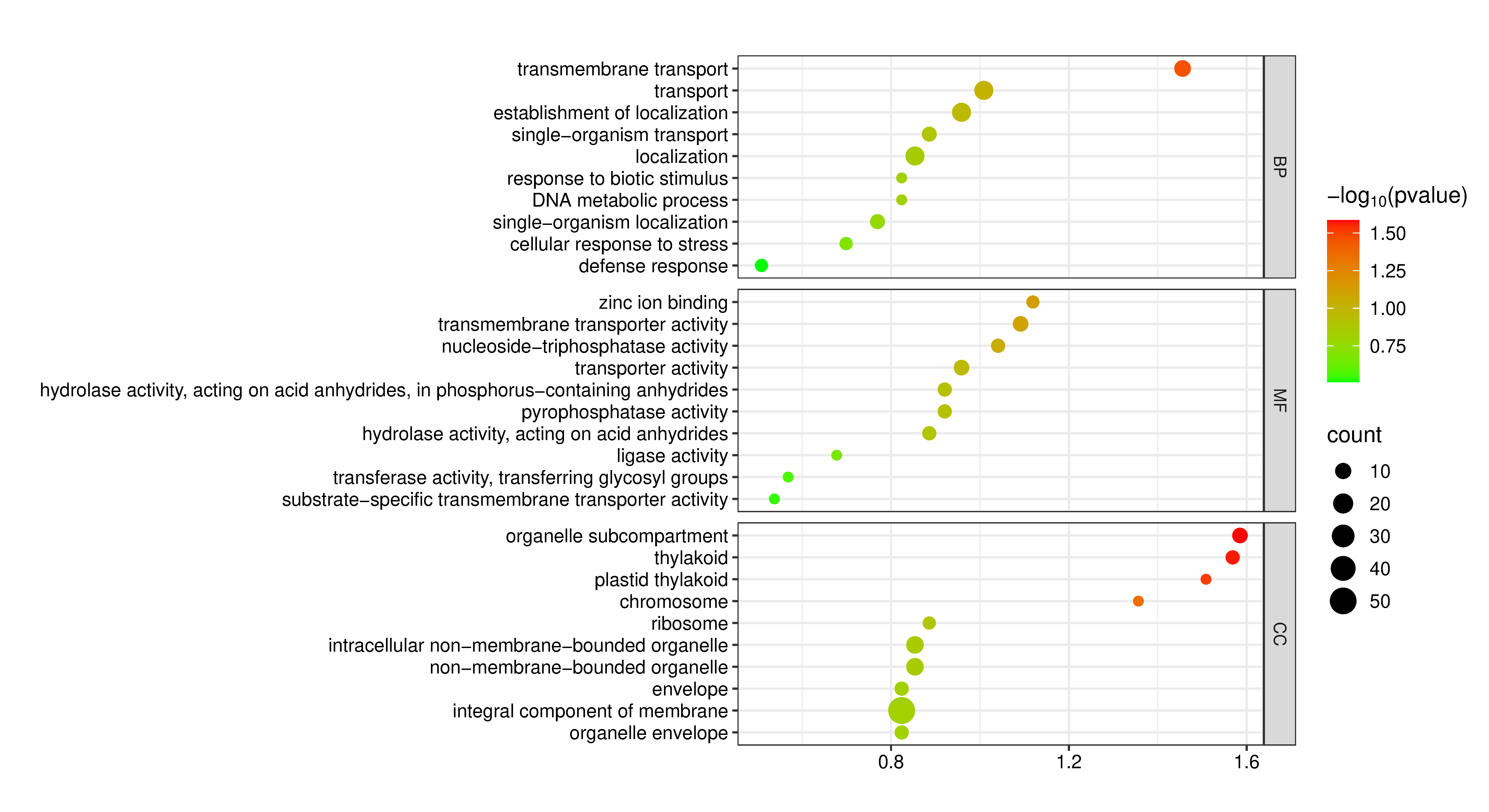


**Supplementary Figure 2** GO enrichment analysis of 122 candidate genes. BP: biological processes; MF: molecular functions; CC: cellular components. Only the top 10 GO terms are displayed. The color of bubbles represents the corrected P value. The size of bubbles represents the number of candidate genes in the GO term.


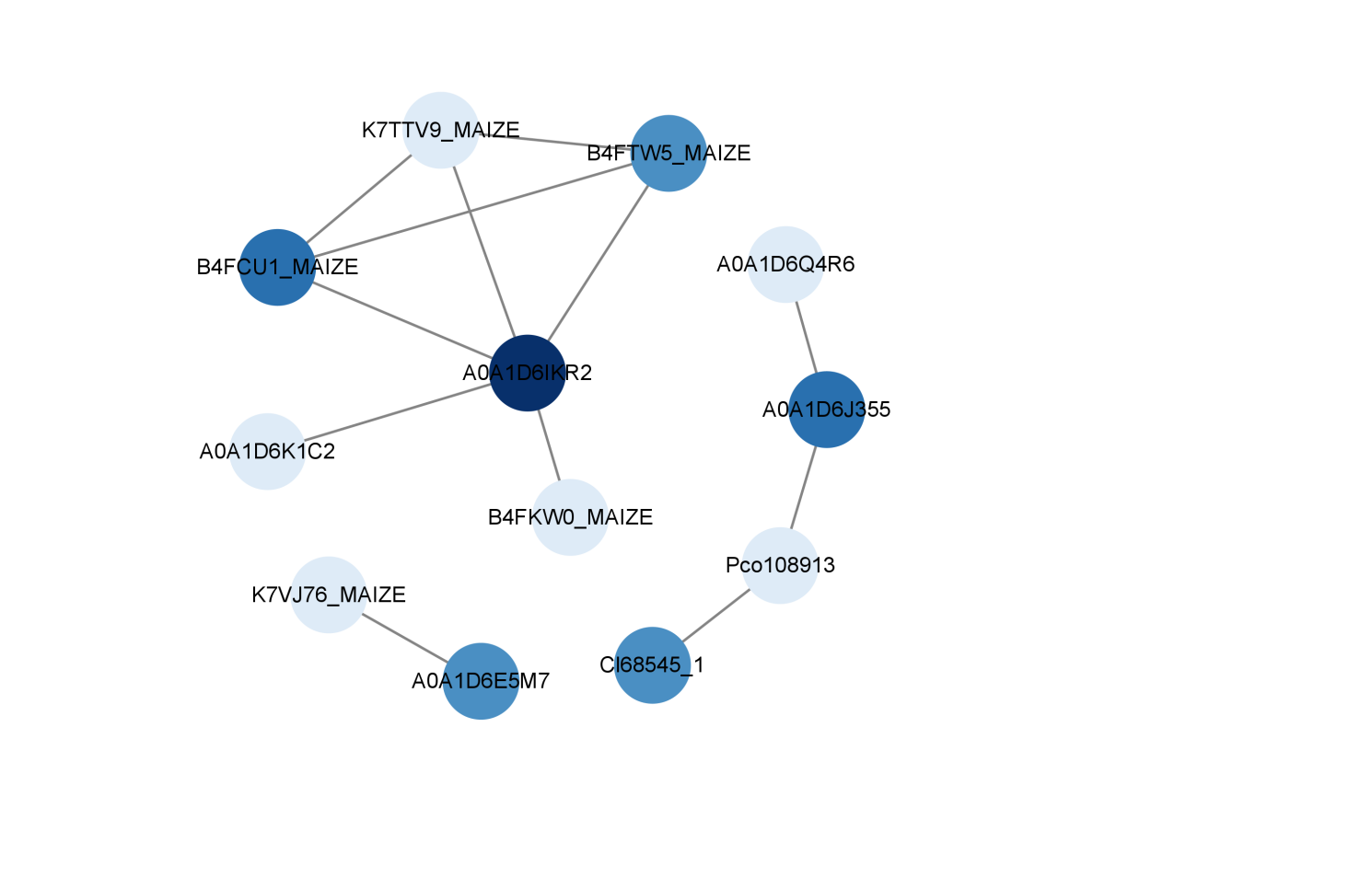


**Supplementary Figure 3** Protein Interaction Network. Twelve genes forming three distinct interaction clusters were identified using the STRING database and Cytoscape software. Different colors represent different levels of interaction intensity, with darker colors indicating stronger interactions.
